# Targeting STAT3 by a small molecule suppresses pancreatic cancer progression

**DOI:** 10.1038/s41388-020-01626-z

**Published:** 2021-01-08

**Authors:** Huang Chen, Aiwu Bian, Lian-fang Yang, Xuan Yin, Jie Wang, Chaowen Ti, Ying Miao, Shihong Peng, Shifen Xu, Mingyao Liu, Wen-Wei Qiu, Zhengfang Yi

**Affiliations:** 1 East China Normal University and Shanghai Fengxian District Central Hospital Joint Center for Translational Medicine, Shanghai Key Laboratory of Regulatory Biology Institute of Biomedical Sciences and School of Life Sciences, East China Normal University, Shanghai, 200241 China; 2 Shanghai Engineering Research Center of Molecular Therapeutics and New Drug Development, School of Chemistry and Molecular Engineering, East China Normal University, Shanghai, 200241 China; 3 Shanghai Municipal Hospital of Traditional Chinese Medicine, Shanghai University of Traditional Chinese Medicine, Shanghai, China

**Keywords:** Pancreatic cancer, High-throughput screening

## Abstract

Pancreatic cancer is lethal in over 90% of cases since it is resistant to current therapeutic strategies. The key role of STAT3 in promoting pancreatic cancer progression has been proven, but effective interventions that suppress STAT3 activities are limited. The development of novel anticancer agents that directly target STAT3 may have potential clinical benefits for pancreatic cancer treatment. Here, we report a new small-molecule inhibitor (N4) with potent antitumor bioactivity, which inhibits multiple oncogenic processes in pancreatic cancer. N4 blocked STAT3 and phospho-tyrosine (pTyr) peptide interactions in fluorescence polarization (FP) assay, specifically abolished phosphor-STAT3 (Tyr705), and suppressed expression of STAT3 downstream genes. The mechanism involved the direct binding of N4 to the STAT3 SH2 domain, thereby, the STAT3 dimerization, STAT3-EGFR, and STAT3-NF-κB cross-talk were efficiently inhibited. In animal models of pancreatic cancer, N4 was well tolerated, suppressed tumor growth and metastasis, and significantly prolonged survival of tumor-bearing mice. Our results offer a preclinical proof of concept for N4 as a candidate therapeutic compound for pancreatic cancer.

## Introduction

Signal transducer and activator of transcription 3 (STAT3) is a key element in multiple signaling pathways. STAT3 is prevalent and active in a variety of human cancers. It promotes tumor progression by promoting tumor cell proliferation, survival, tumor invasion, angiogenesis, and immunosuppression [1–3]. STAT3 activation involves the phosphorylation of a critical tyrosine residue, Tyr705. Phosphorylation of Tyr705 results in homo- or hetero-dimerization of STAT3, enabling nuclear localization and DNA binding. This triggers downstream gene transcription, which regulates fundamental cellular processes. Recent studies have revealed novel and diverse functions of STAT3 that are independent of its classic function in cancer [4]. For example, STAT3 regulates mitochondrial functions to drive malignant transformation in certain forms of cancer [5–7].

Human STAT3 is composed of seven distinct domains, including the N-terminal domain, linker domain, coiled-coil domain, DNA-binding domain, Src Homology 2 domain (SH2), C-terminal domain, and transactivation domain [3, 8]. Of these domains, the SH2 domain is the most conserved and critical. It directly interacts with the phosphorylated STAT3-Tyr705 peptide to achieve STAT3 homo-dimerization and enters the nucleus to regulate the expression of its downstream genes. The SH2 domain is also involved in the interaction between c-Jun N-terminal kinase (JAK) and endothelial growth factor receptor (EGFR), followed by increased phosphorylation of STAT3.

Inhibitors of STAT3 can be divided into direct and indirect groups [9, 10]. Indirect inhibitors target the upstream components of the STAT3 pathway that affect STAT3 protein by decreasing its Tyr705 phosphorylation. In this group, JAK and SRC kinase are the major drug development targets, and many JAK inhibitors have been evaluated in clinical trials for indications, such as oncology and inflammatory syndromes, and ruxolitinib and tofacitinib have been approved by the United States Food and Drug Administration. However, the ongoing clinical trial results have also revealed potential side effects of JAK inhibitors that include increased risks of infection, anemia, and thrombocytopenia [11–13].

Direct STAT3 inhibitors comprise several groups based on the binding domain with STAT3. Current research on direct STAT3 inhibitors is focused on antagonizing the SH2, given its importance in promoting STAT3 activation and subsequent regulation of transcriptional functions. The earliest discovered SH2 inhibitors were phosphopeptide and its peptide mimic. The phosphopeptide has limited membrane permeability and stability, and so is of limited clinical value. Many studies have sought to antagonize STAT3-SH2 with small-molecule inhibitors. A few SH2 inhibitors have shown potential for further clinical application. C188-9 (TTI-101) was developed for the treatment of advanced tumors. It has been evaluated in the clinical phase Ι trial of liver cancer, breast cancer, gastric cancer, and other cancer [14, 15]. OPB-111077 is another high-affinity STAT3 inhibitor. It efficiently inhibits STAT3 activity and directly inhibits mitochondrial oxidative phosphorylation. Clinical experiments have been carried out in a variety of malignant tumors [7, 16].

Pancreatic cancer is a lethal condition with poor outcomes and an increasing incidence. Despite significant advances in surgery and multi-agent chemotherapy for cancer management, it still has a dismal 5-year survival rate of <8%, which has not changed appreciably over the past 40 years [17, 18]. The discovery and development of specifically targeted therapies for pancreatic cancer are needed. STAT3 has emerged as a rationale drug target for pancreatic cancer [19–22]. Persistent activation of STAT3 due to phosphorylation of Tyr705 has been observed in nearly 30–100% of human tumor specimens [23, 24]. The use of conditional knockout mice also proved that STAT3 is not dispensable for pancreas development and homeostasis [25], suggesting its specificity for the pancreatic cancer treatment. STAT3 incorporated with pancreatic ductal adenocarcinoma high-frequency mutations like K-RAS and P53 worsens the disease outcome. Moreover, STAT3 inhibitors, such as the small-molecule inhibitor BBI-608 and monoclonal antibody SBT-100 have obtained orphan drug identification in pancreatic cancer and are undergoing clinical phase ΙΙΙ and Ι study, respectively.

The targeted inhibition of STAT3 represents a promising therapeutic strategy for pancreatic cancer. In this study, we document that N4 is a direct STAT3 inhibitor as demonstrated using fluorescence polarization (FP), surface plasmon resonance (SPR), and microscale thermophoresis (MST) assays. N4 selectively inactivated STAT3 activation, thereby suppressing the growth and migration of pancreatic cancer cells in vitro. In mouse models of pancreatic cancer, N4 suppressed tumor growth, metastasis, and prolonged the survival of tumor-bearing mice. Our data establish N4 as a STAT3 inhibitor that has potential therapeutic value for pancreatic cancer.

## Results

### Discovery of N4

**Fig. 1:**
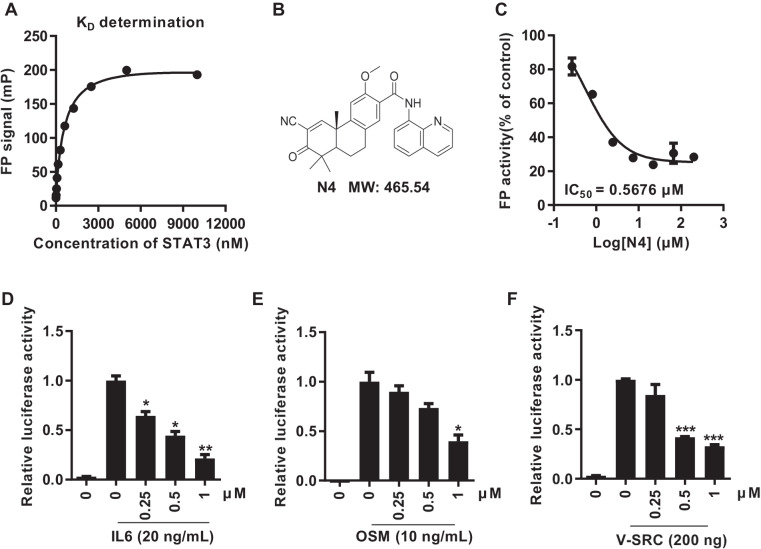
N4 inhibits STAT3 dimerization and transcription in vitro. **A** Purified STAT3 protein was incubated with labeled phosphopeptide, 5-carboxyfluorescein-GpYLPQTV-NH2 for 1 h at 37 °C. FP signals were measured, and the data were analyzed using Graph Pad 7.0 software. **B** Chemical structure of N4. **C** Different concentrations of N4 were pre-incubated with recombined STAT3 protein for 1 h, then 10 nM of labeled peptide was added for 1 h. The IC_50_ was calculated using Graph Pad 7.0. **D**, **E**, **F** STAT3 specific luciferase reporter plasmid was transiently transfected into 293T cells. After 24 h transfection, IL-6 (20 ng/mL), OSM (10 ng/mL), and V-SRC expression plasmid (200 ng) was used to activate STAT3 luciferase activity, N4 was added at the indicated concentrations. Renilla luciferase activities were used as an internal reference (*n* = 2 per group). The values are expressed as mean ± SD and the bars indicate statistically significant differences (*t* test), *P < 0.05; **P < 0.01; ***P < 0.001.

To identify compounds capable of inhibiting the STAT3 SH2 domain, we configured a STAT3-specific FP assay. The use of FP assay for STAT3-SH2 drug screening has been previously reported [26]. We purified amino acids 127–722 of the STAT3 protein for this assay. Following a 1 h incubation of the purified STAT3^127–722^ protein and peptide at 37 ^°^C, purified STAT3 directly interacted with peptide in a concentration-dependent manner (Fig. 1A). Stattic, BP-1-102, and SH-4-54 were reported to inhibit STAT3 by interacting with the SH2 domain. In the FP assay model, the fluorescence signal was suppressed by all these inhibitors in a concentration-dependent manner, with an IC_50_ that was consistent with previous reports (Supplementary Fig. S1). The assay was used to screen our internal chemical library. After several rounds of validation of the screening model, we identified N4 (Fig. 1B, Supplementary Figs. S2, S3, Supplementary Table S1) as a putative compound to block the STAT3-peptide interaction, with an IC_50_ of 0.57 μM (Fig. 1C).

To further test the bioactivity of N4, a luciferase reporter assay was carried out using 293T cells. We first selected a variety of indirect and direct STAT3 inhibitors to confirm the reliability of the assay system. All the positive compounds suppressed luciferase activity with a concentration-dependent manner (Supplementary Fig. S4). Interleukin-6 (IL-6) and Oncostatin-M (OSM) were receptor-dependent activators of STAT3, while, V-SRC activated STAT3 in a receptor-independent manner [27, 28]. In our systems, IL-6, OSM and V-SRC induced STAT3-dependent luciferase reporter activity by 34-, 90-, and 34-fold, respectively, and luciferase activities were suppressed by N4 in dose-dependent manner (Fig. 1D–F). Moreover, the STAT3-response luciferase activities were also examined in several PDAC cells, and N4 efficiently inhibited luciferase activities in PANC-1 and BXPC-3 cells with or without IL-6 stimulation (Supplementary Fig. S5). The configuration and use of the STAT3 SH2 domain specific fluorescence polarization and STAT3-response luciferase reporter assays allowed the screening of N4 as a compound with potent inhibitory activity.

### N4 suppresses STAT3 activation in pancreatic cancer cells

**Fig. 2:**
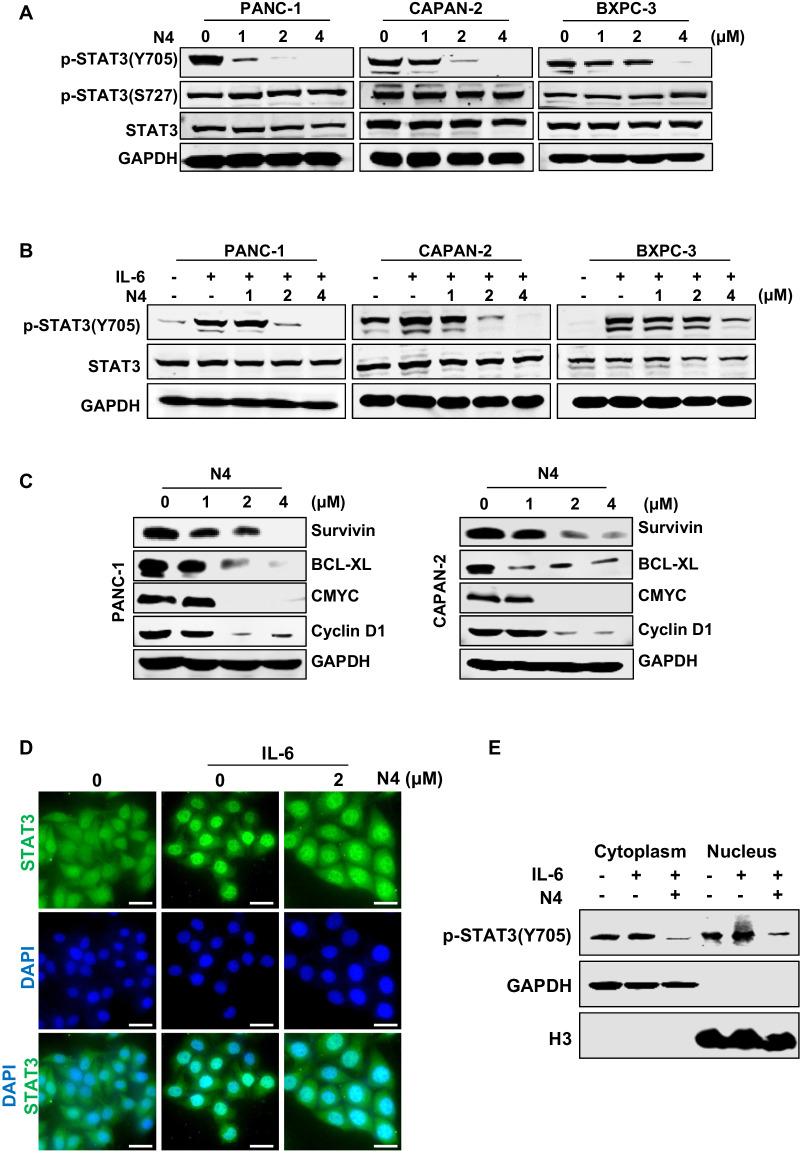
N4 inhibits STAT3 activation in pancreatic cancer cells. **A** PANC-1, CAPAN-2, and BXPC-3 cells were treated with the indicated concentrations of N4 for 24 h. Then, p-STAT3 (Y705), p-STAT3 (S727), and STAT3 were detected by western blot assays with glyceraldehyde 3-phosphate dehydrogenase (GAPDH) used as the loading control. **B** Pancreatic cancer cells were starved in a medium lacking fetal bovine serum (FBS; basic medium) for 24 h, and then pretreated with N4 in basic medium for 24 h. Then, stimulated with IL-6 (20 ng/mL) for 30 min and lysed for western blot analysis using the indicated antibodies. **C** Expression levels of STAT3 downstream genes were detected by western blot following treatment of PANC-1 and CAPAN-2 cells with N4. **D** PANC-1 cells were seeded and allowed to attach, and then pretreated with 2 μM of N4 in FBS-free medium for 24 h. The cells were stimulated for 30 min using IL-6 (20 ng/mL). The STAT3 antibody (green) and 4, 6-diamidino-2-phenylindole (DAPI) (blue) was used for staining and STAT3 and the nucleus, respectively. The samples were photographed and analyzed. The scale bar represents 20 μm. **E** After 24 h treatment with 2 μM N4, PANC-1 cells were stimulated with IL-6 (20 ng/mL) for 30 min, and the cytoplasmic and nuclear extractions were analyzed by western blot. p-STAT3 (Y705) was used to detect STAT3, and GAPDH and H3 were used as the cytoplasm and nucleus internal control, respectively.

To confirm whether N4 was influenced by STAT3 activation in pancreatic cancer cells. PANC-1, CAPAN-2, and BXPC-3 cells were treated with the indicated concentrations of N4. After 24 h, the cells were then lysed for western blot analysis. N4 selectively blocked STAT3 Tyr705 phosphorylation but showed no obvious inhibition on Ser-727 phosphorylation and STAT3 protein expression (Fig. 2A). N4 abrogated IL-6-stimulated STAT3 phosphorylation in pancreatic cancer cells (Fig. 2B). We also evaluated SHP2 (PTPase), phospho (p)-STAT1, p-STAT5, p-extracellular signal-regulated kinase, and p-AKT levels in western blots. N4 showed no obvious influence in the expression of these proteins (Supplementary Fig. S6). Further, the protein levels of STAT3-downstream genes were also measured in PANC-1 and CAPAN-2 cells after N4 treatment. As shown in Fig. 2C, N4 reduced STAT3 target gene expression in a concentration-dependent manner.

The SH2 domain mediates STAT3 dimerization. The dimer is translocated to the nucleus for the following transcriptional regulation. As the nuclear translocation is the critical step for STAT3 activation, we next performed immunofluorescent experiments to verify whether N4 had an impact on STAT3 nuclear translocation. PANC-1 cells were exposed to 2 μM N4 for 24 h, followed by stimulation of the cells with IL-6. As shown in Fig. 2D, IL-6 efficiently stimulated STAT3 entry into the nucleus, and N4 substantially impaired STAT3 nuclear translocation. The subcellular fractionation experiment also proved that N4 abrogated STAT3 translocate into the nucleus (Fig. 2E). Taken together, the findings indicated that N4 specifically decreased STAT3-Tyr705 phosphorylation and STAT3 downstream gene expression, and also abrogated STAT3 entry into the nucleus. Thus, N4 can suppress STAT3 activation in pancreatic cancer cells.

### N4 directly binds to STAT3 SH2 domain

**Fig. 3:**
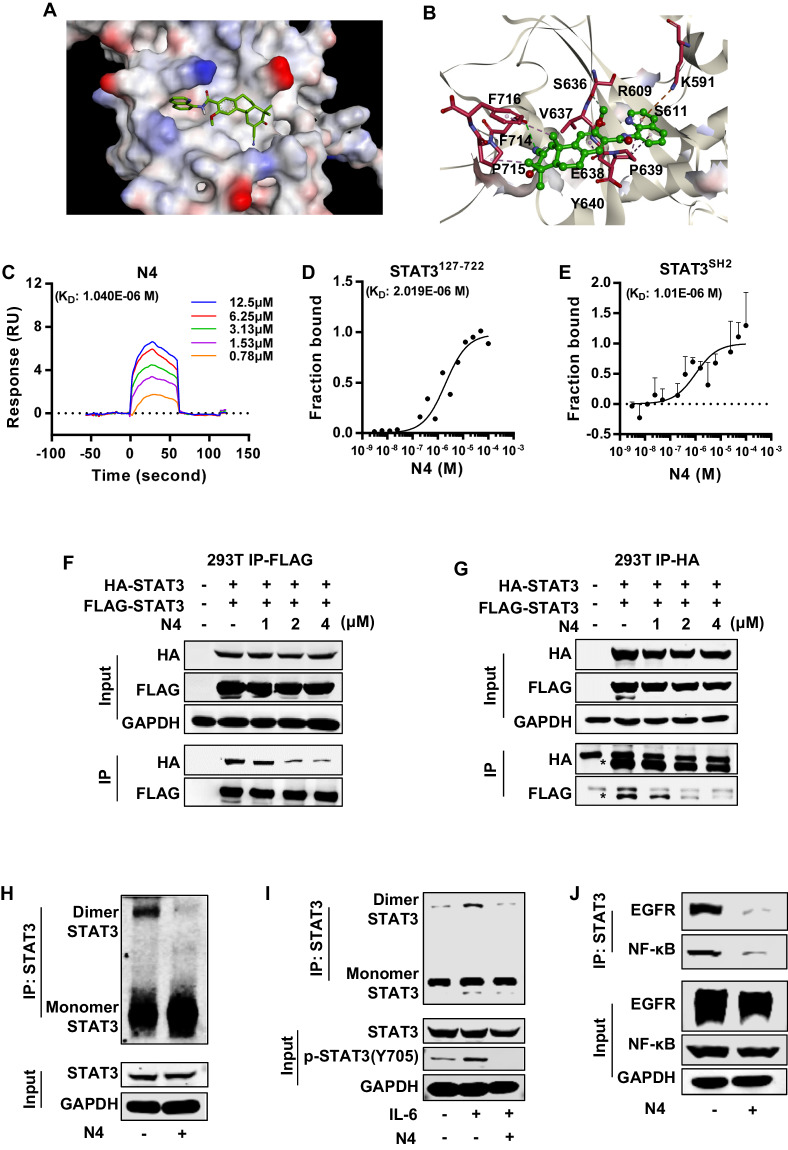
N4 interacts with the STAT3-SH2 domain and interferes with the STAT3-EGFR and STAT3-NF-κB interactions. **A**, **B** Molecular docking model revealed that N4 binds to the SH2 domain of STAT3. **C**–**E** The binding between N4 and STAT3 was examined in (C) SPR and (D) MST experiments. His-STAT3^127-722^ protein was purified and used to examine the affinity between STAT3 and N4. (E) The binding between N4 and STAT3^SH2^ was confirmed in MST assays. STAT3-SH2 protein was purified and labeled. N4 was serially diluted and mixed with equal volumes of labeled STAT3^SH2^ protein. The MST signal was measured, and the data were analyzed by MO analysis software. **F**, **G** The FLAG-tag and HA-tag STAT3 expression plasmid was transfected into 293 T cells, followed by the addition of the indicated concentrations of N4. After 24 h, cells were lysed, and immunoprecipitation experiments were performed as described in Materials and Methods. **H** N4 represses STAT3 dimerization in pancreatic cancer cells. **I** After a 24 h treatment with 2 μM N4, pancreatic cancer cells were stimulated with IL-6 for 30 min. Cell lysates were immunoprecipitated with STAT3 overnight. The precipitates were washed, suspended in non-reducing sample buffer, and boiled for 10 min. The indicated antibodies were used for western blot assays. **J** N4 inhibits STAT3-EGFR and STAT3-NF-κB interaction in pancreatic cancer cells. PANC-1 cells were treated with 2 μM N4 for 24 h. Cell lysates were immunoprecipitated with STAT3 and blotted with the indicated antibodies.

Our data demonstrated that N4 was a potential STAT3 inhibitor and it inhibited STAT3 activation in pancreatic cancer. Then, we performed the docking assays to evaluate whether N4 could directly bound to STAT3. The molecular modeling of N4 in complex with STAT3 was shown in Fig. 3A, and N4 could interact with multiple amino acid residues of STAT3 including Lys591, which was critical for STAT3-SH2 and Tyr705 interaction (Fig. 3B). To further verify whether N4 interacted directly with STAT3-SH2 domain, the SPR and MST assays were performed. In the SPR assay, N4 bound with STAT3^127–722^ in a time-dependent saturation manner with a *K*_D_ value of 1.04 μM (Fig. 3C). Further, a positive SH2 inhibitor (Stattic) was also used in SPR experiments. Stattic bound to STAT3 with a *K*_D_ of 9.62 μM, which was consistent with previous reports (Supplementary Fig. S7). The MST assay is a new technology for the biophysical analysis of interactions between biomolecules. The assay allows the measurement of interactions directly in the solution without the need for immobilization to a surface [29, 30]. Consistent with the SPR results, in the MST assay N4 interacted with STAT3^127–722^ with a *K*_D_ of 2.02 μM (Fig. 3D). Next, we purified STAT3-SH2 for the further binding experiment. N4 interacted with STAT3-SH2 in concentration-dependent manner with a *K*_D_ of 1.01 μM (Fig. 3E). The collective findings confirmed the direct interaction between N4 and the STAT3 SH2 domain. N4 also displayed higher affinity to STAT3 than Stattic.

Cognizant of the importance of STAT3-SH2 in STAT3 dimerization, we further evaluated whether N4 affected the dimerization of STAT3 in cells. The expression plasmid with differently labeled STAT3 (HIS-tag and FLAG-tag) was co-transfected in 293T cells, and immunoprecipitation (IP) was carried out. As shown in Fig. 3F, G, N4 significantly suppressed STAT3 dimerization in concentration-dependent manner. To further evaluate N4 inhibitory effects on STAT3 dimerization in pancreatic cancer cells, a STAT3 dimerization experiment was performed in PANC-1 cells. N4 efficiently decreased STAT3 dimerization at 2 μM with or without IL-6 stimulation (Fig. 3H, I).

STAT3 can complex with a variety of important proteins to redirect multiple signals for oncogenic functions. Given that STAT3 directly interacts with EGFR in a reaction mediated by its SH2 domain [31, 32], we next evaluated the ability of N4 to inhibit the STAT3–EGFR interaction. PANC-1 cells were treated with vehicle or N4 for 24 h, and then the contact cell lysis was performed for Co-immunoprecipitation (Co-IP) experiments as described in Materials and Methods. As shown in Fig. 3J, N4 inhibited the STAT3–EGFR interaction at a concentration of 2 μM. Previous reports demonstrated that the persistent activation of STAT3 directly regulates the activities of NF-κB in the nucleus. When STAT3 is phosphorylated at Tyr705, the interaction between STAT3 and NF-κB in the nucleus is enhanced [33]. Therefore, we hypothesized that once N4 inhibited the phosphorylation of STAT3 by binding with SH2, the interaction between STAT3 and NF-κB would be inhibited. To examine this hypothesis, the STAT3-NF-κB complex was examined in a Co-IP experiment. As results shown, 2 μM N4 blocked the interaction between STAT3 and NF-κB (Fig. 3J). The collective findings indicated that N4 inhibits STAT3 homo- or hetero-dimerization by interfering with the SH2 domain of STAT3.

### N4 suppresses pancreatic cancer cell growth, migration, and induces apoptosis

**Fig. 4:**
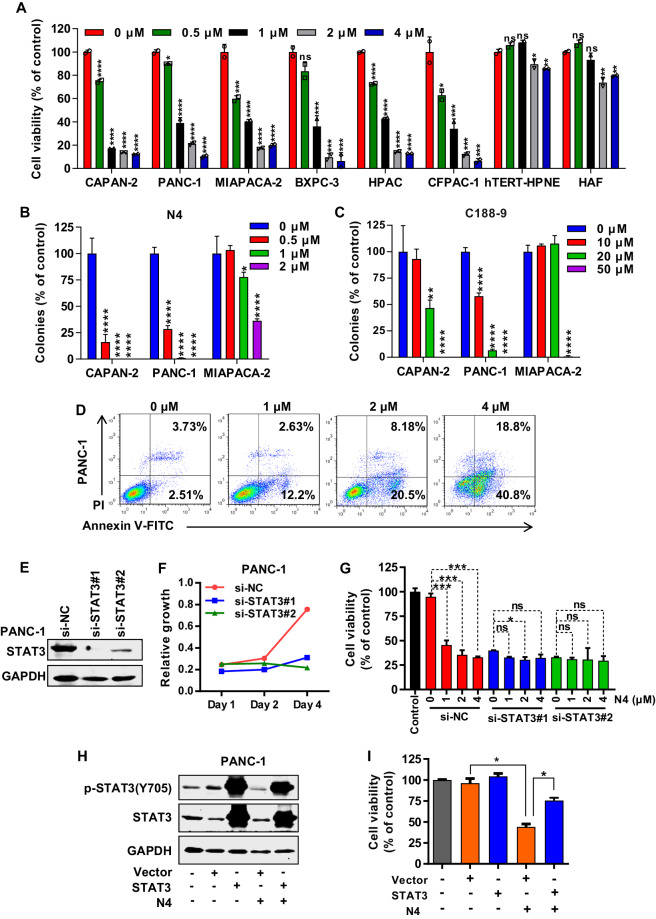
N4 suppresses pancreatic cancer cell proliferation and migration and induces apoptosis. **A** Pancreatic cancer cells (CAPAN-2, PANC-1, MIAPACA-2, BXPC-3, HPAC, and CFPAC-1) and normal cells (HPNE, HAF) were incubated with the indicated concentration of N4 for 48 h. MTS assays were performed to detect the N4-mediated inhibition of cell proliferation. Data are shown as mean ± SD, *P < 0.05, **P < 0.01, ***P < 0.001, ****P < 0.0001, n.s. not significant by one-way ANOVA followed by multiple comparisons. **B**, **C** CAPAN-2, PANC-1, and MIAPACA-2 cells were treated with N4 (B) or C188-9 (C) for 7 days. The cells fixed and stained, and colony numbers were calculated. Experiments were carried out in triplicate with identical results obtained. Data are shown as mean ± SD, *P < 0.05, **P < 0.01, ****P < 0.0001, n.s. not significant by one-way ANOVA followed by multiple comparisons. **D** N4 induces pancreatic cancer apoptosis. Pancreatic cancer cells were treated with the indicated concentrations of N4 for 24 h. Apoptotic cells were analyzed as described in Materials and Methods. **E**–**G** The scrambled siRNA or STAT3 siRNA1# and 2# was transfected in PANC-1 cells. In **E**, the knockdown efficiency was examined. In **F**, after 48 h transfection, transfected cells were seeded in 96-well plates, and cells were enumerated at day 1, day 2, and day 4, In **G**, different cells transfected with different siRNA were treated with the indicated concentrations of N4, and cell viability measured after a 48 h treatment. Data are expressed as mean ± SD, *P < 0.05, ***P < 0.001, n.s. not significant by one-way ANOVA followed by multiple comparisons. **H**, **I** Vector or STAT3 expression plasmids were transfected into PANC-1 cells. 48 h later, 2 μM N4 was added for 24 h, and western blot and MTS assays were performed. Data are expressed as mean ± SD, *P < 0.05, ***P < 0.001, n.s. not significant; Student’s *t* tests were performed.

To further verify the antitumor effects of N4 in pancreatic cancer, the antitumor proliferation activities of N4 were examined. N4 effectively inhibited the proliferation of a variety of pancreatic cancer cells, and had no significant effect on two normal cell types (Fig. 4A). In addition, N4 more potently inhibited several pancreatic cancer cells colony formation than that of C188-9 and BP-1-102 (Fig. 4B, C, Supplementary Fig. S8A, B). N4 also efficiently blocked pancreatic cancer migration (Supplementary Fig. S8C), and induced pancreatic cancer cells apoptosis in a concentration-dependent manner (Fig. 4D). To validate the STAT3 dependency of N4, STAT3 expression was knocked down in PANC-1 cells using small interfering RNA. The knockdown of STAT3 expression significantly affected the proliferation of PANC-1 cells, and the inhibitory effect of N4 was also compromised (Fig. 4E–G, the siRNA sequence listed in Supplementary Table S2). Next, we transiently expressed STAT3 plasmid in PANC-1 cells. Forty-eight hours following transfection, the inhibitory effect of N4 on proliferation in different overexpression groups was performed. The efficiency of overexpression was detected by western blot (Fig. 4H). The efficacy of N4 on the inhibition of proliferation of cancer cells was affected (Fig. 4I). Taken together, these results demonstrated that N4 suppressed pancreatic cancer cell growth and migration, and induced apoptosis. Further knockdown and overexpression rescue experiments proved that STAT3 was the main target of N4.

### N4 causes regression of pancreatic cancer tumors in vivo

**Fig. 5:**
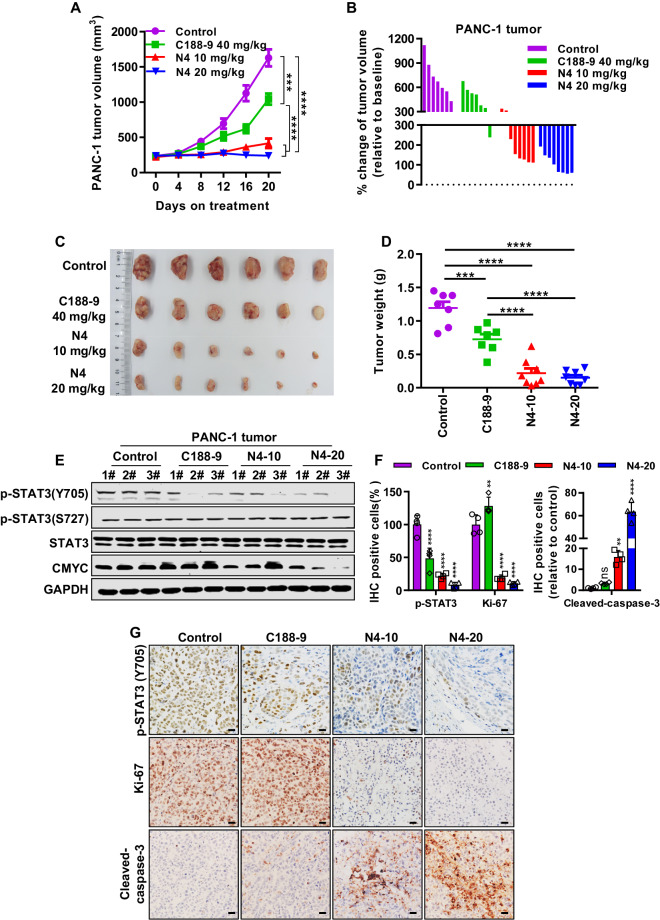
N4 suppresses pancreatic cancer growth in vivo. PANC-1 cells were injected into the right flank of BALB/c-nude mice. After the volume of tumor nodules reached 200–300 mm^3^, the mice were randomly assigned to the indicated groups and were injected intraperitoneally daily with vehicle (*n* = 7), 40 mg/kg/d C188-9 (*n* = 7), 10 mg/kg/d N4 (*n* = 8), or 20 mg/kg/d N4 (*n* = 8). The mice were sacrificed after treatment for 20 days and the tumors from each group of mice were excised. **A**, **B** Tumor volumes were measured every 4 days. Tumor volumes were calculated by the formula: length × width^2^ × 0.52. These data are expressed as mean ± SD; ***P < 0.001 and ****P < 0.0001 by one-way ANOVA. **C** After treatment for 20 days, the tumors from each group of mice were excised and photographed. **D** After the experiments, all the tumors were weighted. These data are expressed as mean ± SD, ***P < 0.001 and ****P < 0.0001 by one-way ANOVA. **E** Tumor samples were collected, and protein was extracted for western blot analysis. p-STAT3 (Y705), p-STAT3(S727), STAT3, and CMYC were detected, with GAPDH used as the loading control. Immunohistochemistry examinations were performed in the different treatment groups, p-STAT3 (Y705), Ki-67 and cleaved caspase3 were quantified (**F**) and photographed (**G**). The scale bar denotes 20 μm. Data are expressed as mean ± SD, **P < 0.01, ****P < 0.0001 and n.s. not significant by one-way ANOVA.

To test the antitumor effect of N4 in vivo, subcutaneous tumor growth xenograft models using PANC-1 was established. When the tumor volume reached 150 to 250 mm^3^, mice were divided into four groups: control, positive control (40 mg/kg/d C188-9), 10 mg/kg/d N4, and 20 mg/kg/d N4. Both doses of N4 significantly inhibited pancreatic cancer growth (Fig. 5A–D). Similarly, the tumor weight in the N4 treatment groups was markedly suppressed compared to the control group (Fig. 5D). N4 displayed a markedly more potent effect on tumor volume and tumor weight than C188-9 (Fig. 5A–D). Determination of weight changes of mice in the four groups revealed that both doses of N4 did not affect the bodyweight of mice (Supplementary Fig. 9A). Hematoxylin and eosin staining of tissue sections of main organs showed that N4 did not produce morphological signs of toxicity (Supplementary Fig. S9B). We next examined whether N4 inhibited pancreatic cancer in vivo mainly through STAT3, we carried out western blot and immunohistochemistry examinations of tumor samples. N4 inhibited STAT3-Tyr705 phosphorylation and downstream gene expression in vivo in the western blot (Fig. 5E), which was corroborated by the immunohistochemistry results (Fig. 5F, G). Moreover, at the higher dose, a significant increase in cleaved caspase 3 was observed, which provided evidence for N4-induced apoptosis in vivo. As additional markers of proliferation, the Ki-67 expression was also examined, and administration of N4 significantly inhibited Ki-67 expression in PANC-1 xenograft model (Fig. 5F, G). The collective findings indicated that N4 can potently inhibit PANC-1 tumor growth and induced apoptosis in vivo, and also abrogate STAT3 phosphorylation and downstream gene expression.

To provide further evidence that STAT3 is the direct target of N4, we constructed two STAT3 stable knockdown cell lines by the short hairpin RNA (shRNA) (Supplementary Fig. S10A), and the sequence of shRNA was list in Supplementary Table S3. As results showed, the shRNA that targets STAT3 but not negative control (NC) shRNA inhibited the growth of PANC-1 cells in vivo (Supplementary Fig. S10B–D); importantly, STAT3 stable knockdown cells were not sensitive to N4 treatment compared with control cells (Supplementary Fig. S10B–D), indicating that STAT3 was a key target of N4. At the meantime, the effect of drug withdrawal on tumor volume and weight was also examined. After 20 days of treatment, all the drug or vehicle were removed and the tumor was allowed to grow for another 12 days. As the results showed, the tumor volume of the vehicle group (shNC-Control) was increased 1086.64 mm^3^ during the drug withdrawal period, while, a slight increase was observed in the N4 treatment group (shNC-N4), with the 249.49 mm^3^ increase in tumor volume (Supplementary Fig. S10C). Thus, N4 kept the suppression on tumor growth compared to vehicle group during the drug withdrawal period.

### N4 suppress pancreatic tumor liver metastasis in vivo

**Fig. 6:**
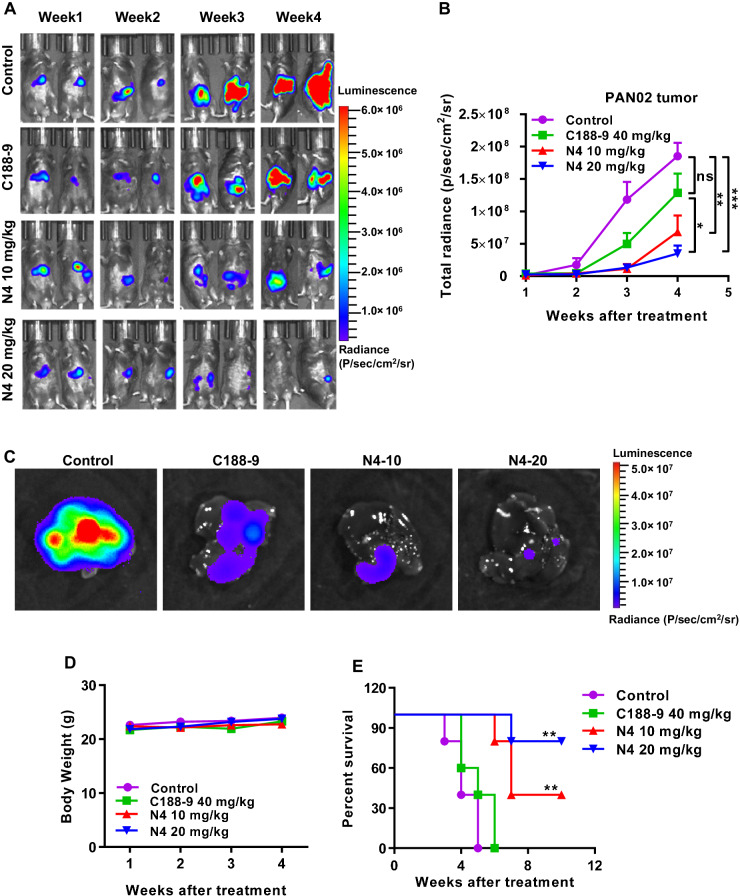
N4 exhibits potent anti-metastatic activity in a pancreatic cancer liver metastasis mouse model, and prolongs survival of tumor-bearing mice. C57BL/6 mice were intrasplenically injected with luciferase-expressing PAN02 cells (PAN02-luc) in matrigel and tumor growth was assessed (**A**) and quantified (**B**) by bioluminescent imaging weekly. Data represent the mean ± SD; *P < 0.05, **P < 0.01, ***P < 0.001, ****P < 0.0001, n.s. not significant by one-way ANOVA followed by multiple-comparison tests. **C** After 4 weeks, mice were sacrificed and livers were imaged. **D** The body weight of C57BL/6 tumor-bearing mice were measured weekly. (*n* = 6 mice per group) **E** Overall survival rate of mice in the different treatment groups (*n* = 5 mice per group). *P < 0.05, **P < 0.01, ***P < 0.001, ****P < 0.0001, n.s. not significant by log-rank (Mantel–Cox) test.

Advanced pancreatic cancer is often accompanied by lethal liver metastasis, which is presently an incurable condition. Our finding that N4 can significantly inhibit pancreatic tumor growth in vivo prompted the examination of whether N4 can inhibit tumor metastasis. This was assessed using a pancreatic liver metastasis model. PAN02 cells expressing luciferase were injected into the spleens of C57BL/6 mice and the tumor metastasis was monitored weekly. The pancreatic tumors quickly metastasized to the livers of the mice (Fig. 6A). Based on their bioluminescence signals, the mice were grouped into a control group, 10 mg/kg/d and 20 mg/kg/d N4 treatment groups, and C188-9 treatment group. Administration of either dose of N4 significantly inhibited liver metastasis of pancreatic cancer, and the anti-tumor metastasis effect was more potent than that of C188-9 treatment (Fig. 6A, B). By week 4, compared with the control group, tumor metastasis was suppressed by 30.65% in the C188-9 group and by 63.25% and 81.16% in the 10 and 20 mg/kg/d N4 groups, respectively (Fig. 6B). At the conclusion of this portion of the experiment, the mice were sacrificed and whole livers were immediately removed for immediate measurement of bioluminescence. The results confirmed the metastasis of pancreatic tumors to the liver in the absence of N4 and the N4 suppression of metastasis (Fig. 6C). Besides, N4 was well tolerated which had no obvious effect on mice bodyweight (Fig. 6D). We hypothesized that N4-mediated suppression of liver metastasis of pancreatic cancer would prolong survival. To assess this, we determined survival rates of mice that received vehicle or drug treatments. Administration of 10 mg/kg/d or 20 mg/kg/d N4 significantly prolonged survival of tumor-bearing mice. By contrast, all mice in the control (*n* = 5) and C188-9 (*n* = 5) groups died by week 5 and 6, respectively. To our surprise, on week 10%, 40% and 80% of the mice in the 10 and 20 mg/kg/d N4 treatment groups were alive (Fig. 6E). The collective findings indicated that N4 could effectively inhibit liver metastasis of pancreatic cancer in the preclinical mouse model, which improved the survival rates of tumor-bearing mice.

### N4 decreases tumor burden in an orthotopic pancreatic cancer model

**Fig. 7:**
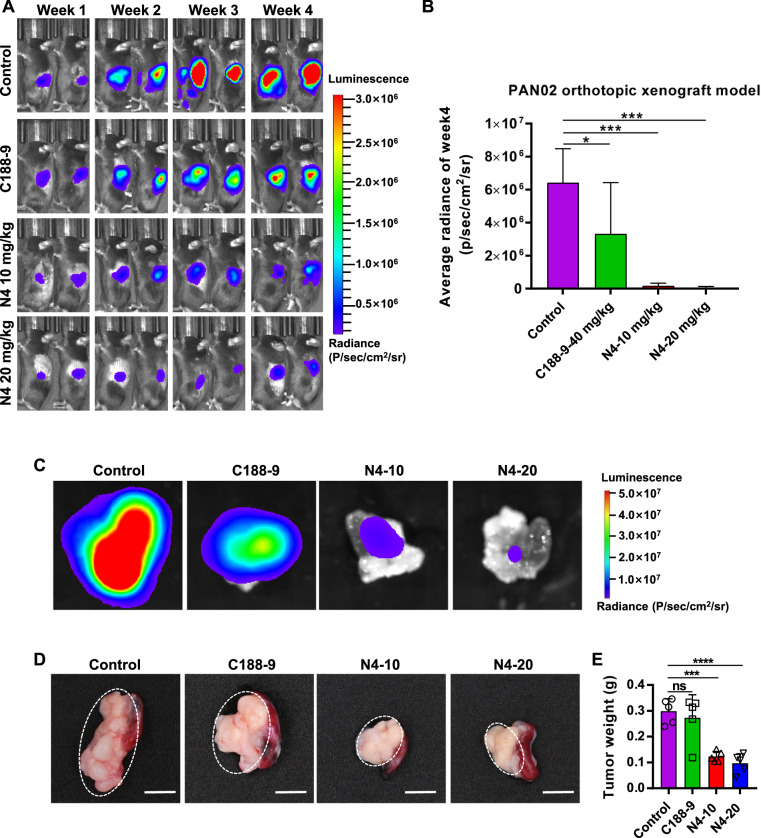
N4 inhibits tumor growth in an orthotopic pancreatic cancer model. C57BL/6 mice were injected the tail of the pancreas with luciferase-expressing PAN02 cells (PAN02-luc) in matrigel and tumor growth was assessed (**A**) and quantified (**B**) by bioluminescent imaging weekly. Data represent the mean ± SD; *P < 0.05, **P < 0.01, ***P < 0.001, ****P < 0.0001, n.s. not significant by one-way ANOVA followed by multiple-comparison tests. (*n* = 5 mice per group) **C** After 4 weeks, mice were sacrificed and the pancreas were imaged. The tumor of pancreas were photographed (**D**) and weighted (**E**), scale bar, 0.5 cm. These data are expressed as mean ± SD, ***P < 0.001, ****P < 0.0001 and n.s. not significant by one-way ANOVA followed by multiple-comparison tests.

The PDAC tissue has the characteristics of high-density stroma to build up the barrier for drug delivery, and the distinctive immunosuppressive microenvironment may also seriously affected the drug response. Thus, it is necessary to evaluate the anti-tumor efficacy of N4 in a pancreatic orthotopic tumor model. We proceeded to test the effect of N4 administration on pancreatic tumors using an orthotopic pancreatic cancer mouse model in which PAN02-luc cells were injected into the tail of the pancreas of C57BL/6 mice. As the results showed, administration of either dose of N4 significantly inhibited tumor growth of pancreatic cancer (Fig. 7A, B). By week 4, compared with the control group, the tumor volume was suppressed by 48.23% in the C188-9 group and by 97.34% and 98.88% in the 10 and 20 mg/kg/d N4 groups, respectively (Fig. 7B). At the end of experiments, mice pancreas were immediately removed, and the bioluminescence and weight of tumor were measured, these results confirmed that N4 was significantly inhibited tumor growth, and the antitumor effect was more effective than that of C188-9 treatment (Fig. 7C–E). Taken together, N4 effectively inhibits pancreatic tumor growth in the orthotopic xenograft models.

### N4 reverses EMT phenotype and shapes the microenvironment of PDAC

**Fig. 8:**
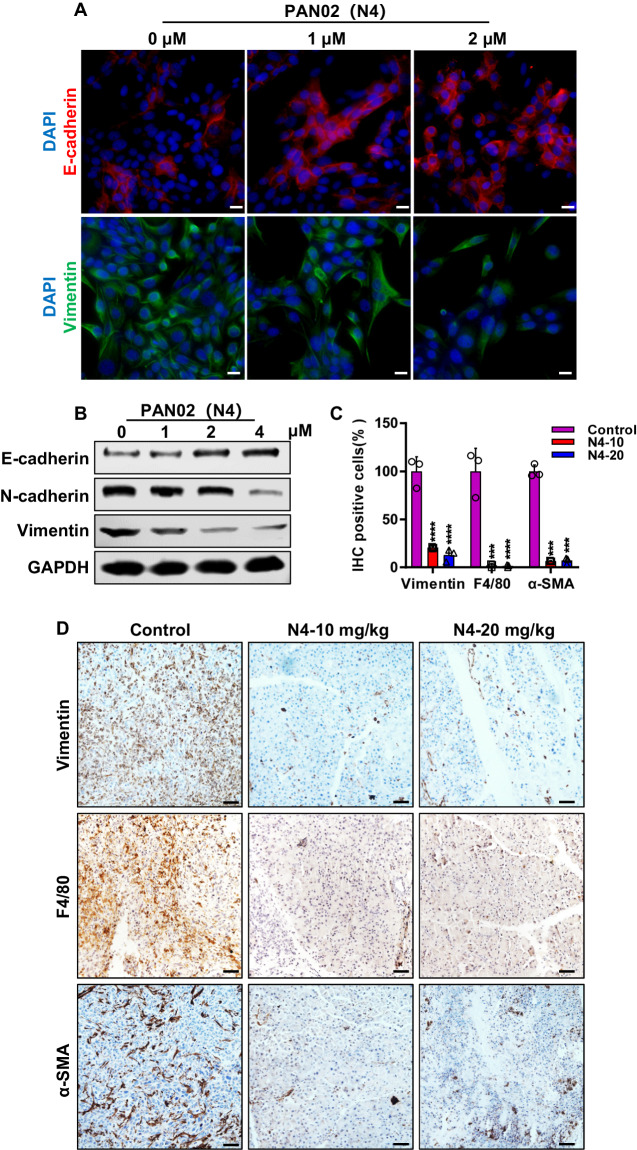
N4 reverses EMT phenotype and shapes the microenvironment of PDAC. **A** IF staining was performed to detected the level of E-cadherin (red) and vimentin (green) after PAN02 treatment with the indicated concentrations of N4 for 24 h. DAPI (blue) was used to stain Nuclei. Scale bar, 20 μm. **B** Western blot assays were used to examine the expression of E-cadherin, N-cadherin and vimentin after PAN02 treatment with N4 for 24 h. **C** IHC of vimentin, F4/80 and α-SMA in tumor tissue from orthotopic models. Data represents the mean ± SD; *P < 0.05, **P < 0.01, ***P < 0.001, ****P < 0.0001, n.s. not significant by one-way ANOVA followed by multiple-comparison tests. **D** Quantification of vimentin, F4/80 and α-SMA in pancreatic tumors from PDAC. Scale bar, 50 μm.

To gain insight into the mechanism of N4 significantly inhibiting tumor growth and metastasis in vitro and in vivo, we first explored how N4 suppressed the pancreatic tumor metastasis. For the Epithelial–Mesenchymal Transition (EMT) is an initial step in the process of tumor metastasis, we detected the epithelial and mesenchymal markers in vitro by immunofluorescence (IF) and WB assays, and N4 markedly lead to the upregulation of E-cadherin, and downregulation of N-cadherin and vimentin in Pan02 cells (Fig. 8A, B). Consistent with the in vitro results, the expression of vimentin was also significantly suppressed in vivo (Fig. 8C, D). The persistent STAT3 activation supports a protumorigenic microenvironment in PDAC by increasing the levels of immuno-suppressive cells, such as tumor-associated macrophages (TAM). Thus, we detected the numbers of the F4/80+ macrophages in pancreatic orthotopic tumor model upon N4 treatment, and administration of N4 significantly decreased the number of TAM (Fig. 8C, D). The microenvironment of PDAC is composed not only of immune cells but also of desmoplastic stroma, and STAT3 was involved in regulating desmoplasia and stromal response. We hypothesized that inhibition STAT3 would result in remodeling of desmoplastic stroma in pancreatic tumors. Indeed, the expression of stromal cells marker, α-SMA, was drastically suppressed in vivo (Fig. 8C, D). Taken together, these data suggest that N4 reversed EMT phenotype in vitro and in vivo, and remodeled pancreatic tumor environment in vivo by decreasing the number of TAM and stromal cells.

## Discussion

Pancreatic cancer is an incurable malignancy and profoundly resistant to all forms of therapy. Novel and effective therapeutic approaches are urgently needed. Numerous studies have shown that STAT3 is an effective drug target for the treatment of pancreatic cancer. However, inhibiting STAT3 with a small-molecule inhibitor has been challenging [34]. Here, we identified a novel STAT3 inhibitor, N4, using FP and STAT3 luciferase reporter screening system, and proved that N4 was directly bound to STAT3 via in silico and in vitro assays. The Stattic, C188-9 and BP-1-102 were the established STAT3 inhibitors, while, N4 exhibited more potency in suppressing pancreatic cancer growth and STAT3 signaling in vitro and in vivo. N4 also selectively inhibited STAT3 activation in pancreatic cancer cells, while displayed little impact on STAT1 and STAT5, which shared the most conserved structure features to STAT3 among STAT members. Moreover, we demonstrated that N4 exhibited potent antitumor activities in pancreatic cancer cells and mouse xenografts models through inhibiting STAT3 signaling.

The SH2 was the most critical and conserved domain of STAT3, and involved in STAT3 dimerization and upstream kinas recognition. When STAT3 Tyr705 was phosphorylated, STAT3 formed dimer and subsequently translocated into the nucleus and triggered the following transcription. On the basis of FP assay results of that N4 displaced the specific STAT3 SH2 binding peptide in vitro, we speculated that N4 could directly bind to STAT3 SH2. Our molecular docking results give further support for this suggestion. Indeed, docking results confirmed that N4 interacted with STAT3-SH2 at multiple amino acid residues including Lys591, Ser636, Val637, and Glu638. Subsequently, we confirmed that N4 directly binds to STAT3-SH2 using the SPR, and MST assays. Moreover, N4 showed approximately tenfold improvement of binding affinities to STAT3 than Stattic in FP and SPR experiments.

One of important finds of this study is that N4 significantly suppressed pancreatic cancer liver metastasis. Pancreatic liver metastasis represents a significant therapeutic challenge and evidence had been provided that STAT3 is involved in key steps of pancreatic cancer liver metastasis, including metastatic niche formation and tumor growth [35–37]. Presently, the administration of N4 potently suppressed pancreatic tumor liver metastasis and significantly prolonged the overall survival of mice with pancreatic cancer tumors. These results indicate the therapeutic potential of N4 for pancreatic cancer that has metastasized to the liver.

Indeed, we also found that overexpression STAT3 cannot fully restore pancreatic cancer cell proliferation from N4 treatment, further studies are needed to determine whether N4 can suppress pancreatic cancer by targeting other tumor-related targets. In addition, our results proved that STAT3- NF-κB interaction was interrupted. Owning to the key role of STAT3 and NF-κB in the regulation of the tumor microenvironment, further investigations also needed to explore whether N4 directly influences the pancreatic tumor microenvironment. Such findings, including the present findings, will deeper the understanding of the mechanism of action of N4.

## Materials and methods

### Chemistry

N4 was synthesized as described in supporting information (Supplementary Figs. S1 and S2). And the Chemical structure of N-series family, Structure–activity relationship for the derivative analogs and IC_50_ value for compounds inhibited the STAT3 dimerization in FP assay were shown in Supplementary Table 1.

### Cell culture

The PANC-1, BXPC-3, CAPAN-2, MIAPACA-2, HPAC, CFPAC-1, PAN02, HAF, hTERT-HPNE, and 293T cell lines were purchased from ATCC and Chinese Academy of Sciences Cell Library. PANC-1, PAN02, MIAPACA-2, HAF and 293T cells were cultured in DMEM (Gibco). BXPC-3, CAPAN-2, and HPAC cells were cultured in RPMI-1640 (Gibco), CFPAC-1 cells were cultured in IMDM (Gibco). hTERT-HPNE cells were cultured in 75% DMEM lacking glucose and 25% medium M3 base and supplemented with 5% fetal bovine serum (FBS), 10 ng/ml human recombinant epidermal growth factor, 5.5 mM D-glucose, and 750 ng/ml puromycin. Except for hTERT-HPNE cells, the media used for the cells was supplemented with 10% FBS (Gibco) and 1% penicillin/streptomycin. The medium for MIAPACA-2 cells also contained 5% horse serum. All the cells were using standard culture conditions at 37 °C in an atmosphere of 5% CO_2_.

### Fluorescence polarization assay

The FP assay was performed as previously described [38]. The labeled phosphopeptide: 5-carboxyfluorescein-GpYLPQTV-NH2 (where pY represents phospho-Tyr) was used as the STAT3 binding probe. To evaluate the effect of compounds in inhibiting STAT3-peptide interaction, serial dilutions of the STAT3 competitors were prepared. The diluted compounds were added to the reaction mixture in 100 μl of FP buffer (50 mM HEPES, pH 7.5, 150 mM NaCl, 0.1% NP-40, and 1 mM dithiothreitol) containing 500 nM his-STAT3^127–722^ protein for pre-incubation at 37 °C for 1 h. Then, 10 nM of the labeled peptide was added and incubated for 1 h. After incubation, FP was measured on a Cytation^TM^ 5 cell imaging multi-mode Reader (BioTek) using excitation and emission wavelengths of 480 nm and 535 nm, respectively. Each reaction was repeated two or three times. Binding or competitive parameters were calculated from a nonlinear regression using GraphPad Prism 7.0.

### Dual luciferase assay

Dual luciferase assay was performed as previously described [39]. STAT3 response luciferase plasmid and Renilla luciferase vector (phRL-TK, Promega) were transiently transfected into 293T cells and PDAC cells (PANC-1 and BXPC-3) using Lipofectamine 2000 (Invitrogen). After 24 h, the transfected cells were treated with indicated compounds for 24 h with or without activator (IL-6, OSM, or V-SRC). Renilla and firefly luciferase activities were determined by luminometry using the Dual Luciferase Reporter Assay System (Promega). The ratio was calculated.

### Surface plasmon resonance assay

Surface plasmon resonance (SPR) analysis was conducted with a Biacore T200 instrument (GE Healthcare) with CM5 sensor chip (GE Healthcare). To test N4 binding of STAT3^127–722^ protein, serially diluted concentrations of N4 were injected into the flow system. Experiments were conducted using phosphate buffered saline (PBS) and the analyte was injected at the flow rate of 30 μL/min. The association time was 60 s and the dissociation time was 30 s. Since N4 was dissolved in PBS containing 5% dimethyl sulfoxide and a solvent correction assay was performed to adjust the results. STAT3^127–722^ protein was immobilized on the sensor chip (CM5) using the amine-coupling method according to standard protocols. N4 or Stattic at various concentrations were injected into the flow system. The kinetics and affinity assay were examined at 25 °C at a flow rate of 30 μL/min. The *K*_D_ values were calculated with the kinetics and affinity analysis option of Biacore T200 plus evaluation software (version 3.0).

### Microscale thermophoresis assay

The MST assay was previously described [40]. Binding affinities of N4 with the purified His-STAT3-SH2 were measured using the Monolith NT.115 device (NanoTemper Technologies). The proteins were fluorescently labeled using the monolith his-tag labeling kit (NanoTemper Technologies) according to the manufacturer’s procedure and kept in an assay buffer comprised of 50 mM HEPES, pH 7.4, 150 mM NaCl, and 0.1% (w/v) Nonidet P-40 at a concentration of 200 nM. The labeled protein was mixed with the same volume of N4 with 16 different serially diluted concentrations at room temperature. The samples were then loaded into premium capillaries (NanoTemper Technologies) and measured using the Monolith NT.115 device. Each assay was repeated two or three times. Data analyses were performed using MO Affinity Analysis v.2.2.4 software and the figures were made by GraphPad Prism 7.0.

### Cell viability

The 3-(4, 5-dimethylthiazol-2-yl)-2, 5-diphenyltetrazolium bromide (MTS) assay was performed to detect cell viability. Briefly, cells were seeded into 96-well plates at the appropriate cell densities. After an overnight incubation to allow cell attachment, the cells were treated with indicated concentrations of the compound for 48 h. MTS was added and, after incubation, the optical density values were measured at 490 nm. The IC_50_ was calculated using GraphPad 7.0 software.

### Colony formation

Pancreatic cancer cells were incubated with the indicated concentration of compounds for 7 days and then the cells were fixed with 4% paraformaldehyde and stained with 0.2% crystal violet. Colonies were photographed, enumerated, and analyzed. Triplicate wells were used for each concentration.

### STAT3 siRNA assay

PANC-1 cells were seeded in six-well plates and transfected with STAT3 siRNA using Lipofectamine 2000 (Thermo Fisher Scientifific, USA). After transfection for 24 h, cells were trypsinized, seeded in 96-well plates and treated with the indicated concentrations of N4. Cell viability was measured using MTS as described above. At the same time, the knockdown efficiency was examined by western blot analysis. The gene-specific siRNAs are listed in Supplementary Table 2.

### Western blotting

Western blotting was performed as previously described [39]. Briefly, cells were treated with indicated compounds and then lysed by boiling. Lysates were immunoblotted with antibodies against STAT3 (9139; Cell Signaling Technology), p-STAT3 (Y705, 9145; Cell Signaling Technology), p-STAT3 (S727, 9134; Cell Signaling Technology), CMYC (ab32072; Abcam), Cyclin D1 (ab134175; Abcam), Survivin (2808; Cell Signaling Technology), MMP2 (4022; Cell Signaling Technology), BCL-XL (2764; Cell Signaling Technology), hemagglutinin (M20003; Abmart), FLAG (F1804; Sigma-Aldrich), EGFR (4267; Cell Signaling Technology), NF-κB (4764; Cell Signaling Technology), E-cadherin (14472; Cell Signaling Technology), N-cadherin (13116; Cell Signaling Technology), vimentin (CY5134; Abways) and GAPDH (ab181602; Abcam). The secondary antibody was conjugated with IRDye 680/800 (926-32221, 926-32210; Millennium Science).

### Co-immunoprecipitation assay

Co-IP was performed as previously described [41]. Pancreatic cancer cells were treated with N4. After 24 h, cells were lysed in IP buffer comprised of 50 mM Tris–HCl, pH 7.4, 0.1% Triton X-100, 1 mM EDTA, 100 mg/ml phenylmethylsulfonyl fluoride, 150 mM NaCl, and protease inhibitor cocktail. The lysates were incubated with STAT3 antibody (9139; Cell Signaling Technology). After overnight incubation, the precipitates were washed and analyzed using western blotting.

### Immunofluorescence staining

Immunofluorescence staining was performed as previously described [42]. Briefly, cells were seeded on gelatin-coated-glass cover slips. After attachment overnight, cells were treated with N4. The cells were fixed, permeabilizated, and blocked using 0.5% bovine serum albumin. The primary antibody including STAT3 (9139; Cell Signaling Technology), vimentin (CY5134; Abways), E-cadherin (14472; Cell Signaling Technology), was incubated at 4 °C. After that, the secondary antibody was incubated. 4′, 6-Diamidino-2-phenylindole was used to stain nuclei of cells. Images were recorded by confocal microscopy (Leica).

### Immunohistochemistry

Briefly, specimens of tumor tissue from xenograft models and orthotopic models were fixed with 4% paraformaldehyde solution at 4 °C overnight. Sections were stained overnight with antibodies against p-STAT3 (Y705, 9145; Cell Signaling Technology), vimentin (CY5134; Abways), α-SMA (BS70000, Bioworld), F4/80 (70076; Cell Signaling Technology), Ki-67 (ab15580; Abcam) at 4 °C, and they were performed using anti-rabbit or anti-mouse secondary antibody. Then, according to the manufacturer’s instructions (SK-4100; Vector laboratories), the avidin-biotin peroxidase complex was used, followed by colorimetric detection using DAB. Finally, hematoxylin was used to counterstain the sections and coverslips were mounted.

### Nuclear protein extraction

Cells were lysed in a hypotonic buffer (10 mM HEPES, 1.5 mM MgCl_2_, 1 mM KCl, 1 mM dithiothreitol, and protease and phosphatase inhibitors), followed by the addition of 0.1% Nonidet P-40. After centrifugation, the cytoplasm protein in the supernatant and nuclear pellets were suspended in the high-salt buffer (hypotonic buffer plus 400 mM NaCl). Protein concentrations were determined, and western blotting was performed.

### In vivo subcutaneous tumor growth xenograft models

BALB/c-nude, male, 6–8-week-old mice were obtained from the Animal Center of East China Normal University. All animal experimental protocols were approved by the Animal Investigation Committee of the Institute of Biomedical Sciences, East China Normal University. PANC-1 cells (5 × 10^6^) were suspended in PBS with 50% Matrigel and injected into the right flank of the mice. Treatment began after the tumor nodules reached 150–250 mm^3^ in volume. The tumor-bearing mice were randomly assigned to four groups and treated with the indicated compounds. The tumor volume and mice body weight were measured after 4 days. The tumor volume (V) was calculated as length × width × width × 0.52. At the end of experiment, the mice were sacrificed. Solid tumors were removed and processed for immunohistochemistry analysis.

### Pancreatic cancer liver metastatic mouse model

C57BL/6, male, 8-week-old mice were obtained from the Animal Center of East China Normal University. All animal experimental protocols were approved by the Animal Investigation Committee of the Institute of Biomedical Sciences, East China Normal University. PAN02 cells that stably expressed luciferase (PAN02-luc) were embedded in Matrigel (BD Biosciences) and intrasplenically injected into mice. Tumors were allowed to grow for 1 week and treatment of the mice with either compound or control was started after grouping of the mice according to average bioluminescence. The IVIS Imaging System (Xenogen Corporation) was used to monitor pancreatic tumor growth and metastasis. Images and measurements of bioluminescent signals were acquired and analyzed using Living Image and Xenogen software.

### Orthotopic pancreatic cancer tumor model

C57BL/6 mice, male, 4–6 week old were obtained from the Animal Center of East China Normal University. All animal experimental protocols were approved by the Animal Investigation Committee of the Institute of Biomedical Sciences, East China Normal University. PAN02 cells that stably expressed luciferase (PAN02-luc) were embedded in Matrigel (BD Biosciences) were injected with 5 × 10^5^ in the tail of the pancreas. On the next day (day 0), the mice were subjected to bioluminescent imaging and divided into four groups according to the luciferase luminescence value. Mice were treated with intraperitoneal injection of indicated compound or drug. Tumor growth was analyzed once a week by an IVIS Imaging System (Xenogen Corporation, Alameda, CA) and end of study tumor weight was determined immediately postmortem. When the mice showed near-death indicators such as loss of mobility and body temperature drop, they were euthanized immediately in consideration of animal ethics. Images and measurements of bioluminescent signals were acquired and analyzed using Living Image and Xenogen software.

### Statistical analyses

Experiments were carried out with three or more replicates. Statistical analyses were done by Student’s *t* test. *P* values < 0.05 were considered significant. The differences between the control group and experimental groups were determined by one-way ANOVA. Data are expressed as mean ± SD and *P* < 0.05 was considered significant. All analysis was performed using Microsoft Excel 2010 and GraphPad Prism 7 software. The animal experiment sample size was chosen to ensure adequate statistical power (>80%) according to formal power calculation.

### Supplementary information

Supporting information

All of the co-authors email responses for the manuscript authorship

## Data Availability

The authors declare that the data supporting this study are available within the paper and its Supplementary Information File. All other data are available from the authors upon reasonable request.
