# Correction: Targeting STAT3 by a small molecule suppresses pancreatic cancer progression

**DOI:** 10.1038/s41388-024-03044-x

**Published:** 2024-06-05

**Authors:** Huang Chen, Aiwu Bian, Lian-fang Yang, Xuan Yin, Jie Wang, Chaowen Ti, Ying Miao, Shihong Peng, Shifen Xu, Mingyao Liu, Wen-Wei Qiu, Zhengfang Yi

**Affiliations:** 1 East China Normal University and Shanghai Fengxian District Central Hospital Joint Center for Translational Medicine, Shanghai Key Laboratory of Regulatory Biology Institute of Biomedical Sciences and School of Life Sciences, East China Normal University, Shanghai, 200241 China; 2 Shanghai Engineering Research Center of Molecular Therapeutics and New Drug Development, School of Chemistry and Molecular Engineering, East China Normal University, Shanghai, 200241 China; 3 Shanghai Municipal Hospital of Traditional Chinese Medicine, Shanghai University of Traditional Chinese Medicine, Shanghai, China

**Keywords:** Pancreatic cancer, High-throughput screening

Correction to: *Oncogene* 10.1038/s41388-020-01626-z

The authors have noticed that an error in Fig. 6A. In the week 2 panel, one of the mice from the N4 10 mg/kg group was inadvertently duplicated with a mouse from the C188-9 group in week 2. Figures 6A have now been corrected as shown below. The authors confirm the corrections do not affect the results or conclusions of this work, and wish to apologies for any inconvenience caused by these inadvertent errors.
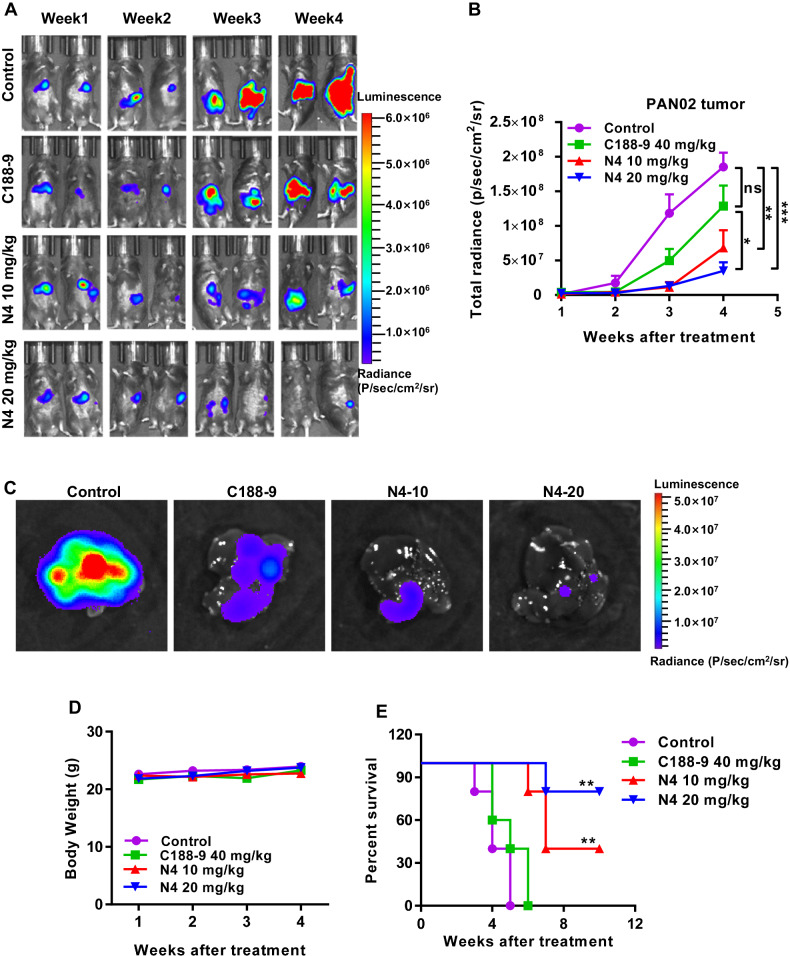


The original article has been corrected.

