# Supplementary material for: Targeting STAT3 by a small molecule suppresses pancreatic cancer progression
Source: Oncogene. 2021 Jan 8;40(8):1440–57. doi: 10.1038/s41388-020-01626-z (PMC7906907; doi:10.1038/s41388-020-01626-z)
Supplement: Supplementary file 1 — Supporting information [file 41388_2020_1626_MOESM1_ESM.pdf]

# Supplementary information

## Supplementary methods:

**Synthesis and spectrograms of N4.** To a solution of compound W318 (10 g, 31.4 mmol) in MeOH 80 mL was added $H_2SO_4$ (1.7 mL, 31.4 mmol). The reaction mixture was heated to reflux for 2 h and then concentrated. Water 30 mL was added to the residue and extracted with EA (30 mL×3). The combined organic extract was washed with brine, dried over $Na_2SO_4$ and concentrated. Purification by silica gel chromatography (petroleum ether/EA, 3:1 v/v) to give compound 5 (9.4 g, 90%) as a white solid. 1H NMR (400 MHz, $CDCl_3$) δ 7.50 (s, 1H), 6.82 (s, 1H), 3.86 (s, 6H), 3.31 (dd, J = 11.2, 5.2 Hz, 1H), 2.92 (dd, J = 16.0, 6.0 Hz, 1H), 2.85 – 2.74 (m, 1H), 2.31 – 2.26 (m, 1H), 1.93 – 1.71 (m, 4H), 1.62 – 1.54 (m, 2H), 1.30 (dd, J = 12.0, 2.0 Hz, 1H), 1.20 (s, 3H), 1.08 (s, 3H), 0.90 (s, 3H).

A solution of compound 5 (9.4g, 28.3 mmol)and IBX (11.9 g, 42.4 mmol) in DMSO 60 mL was stirred at room temperature for 8 h and quenched with $H_2O$ and EA 50. The two layers were separated after filtration and the aqueous was extracted twice with EA. The combined organic extracts were washed with water, rinsed with brine, dried over $Na_2SO_4$ and concentrated under reduced pressure. Purification by silica gel chromatography (petroleum ether/EA, 2:1 v/v) to give compound 6 (8.2 g, 88%) as a white solid. 1H NMR (400 MHz, $CDCl_3$) δ 7.52 (s, 1H), 6.82 (s, 1H), 3.87 (s, 6H), 2.93 (dd, J = 16.8, 4.4 Hz, 1H), 2.86 – 2.57 (m, 3H), 2.49 – 2.43 (m, 1H), 2.03 – 1.97 (m, 1H), 1.91 (dd, J = 12.0, 2.0 Hz, 1H), 1.87 – 1.70 (m, 2H), 1.31 (s, 3H), 1.17 (s, 3H), 1.14 (s, 3H).

A solution of compound 6 (8.2 g, 24.8 mmol) in ethyl formate 60 mL was stirred at 0 °C for 10 mins. Then NaH (4.96 g, 124 mmol) was added under $N_2$. The reaction mixture was stirred at room temperature for 1 h and quenched with water dropwise in 0 °C and was extracted with EA (50 mL×3). The combined organic extracts were washed with water, rinsed with brine, dried over $Na_2SO_4$ and concentrated under reduced pressure. Purification by silica gel chromatography (petroleum ether/EA, 2:1 v/v) to give compound 7 (7.0 g, 79%) as a white solid. 1H NMR (400 MHz, $CDCl_3$) δ 7.50 (s, 1H), 6.81 (s, 1H), 4.55 (dd, J = 12.0, 4.8 Hz, 1H), 3.86 (s, 3H), 3.86 (s, 3H), 2.92 (dd, J = 16.4, 6.0 Hz, 1H), 2.84 – 2.75 (m, 1H), 2.33 – 2.25 (m, 1H), 1.92 – 1.87 (m, 2H), 1.85 – 1.64 (m, 3H), 1.39 (dd, J = 12.0, 1.6 Hz, 1H), 1.22 (s, 3H), 0.97 (s, 3H), 0.96 (s, 3H).

To a solution of compound 7 (7.0 g, 19.5 mmol) in EtOH 80 mL was added Hydroxylamine hydrochloride (6.76 g, 97.5 mmol). The reaction mixture was heated to reflux under N2 for 4 h and then concentrated. Water 40 mL was added to the residue and extracted with EA (40 mL×3). The combined organic extract was washed with brine, dried over $Na_2SO_4$ and concentrated. Purification by silica gel chromatography (petroleum ether/EA, 3:1 v/v) to give compound 8 (6.7 g, 90%) as a white solid. 1H NMR (400 MHz, CDCl3) δ 8.12 (s, 1H), 7.53 (s, 1H), 6.89 (s, 1H), 4.35 (q, J = 7.2 Hz, 2H), 3.90 (s, 3H), 3.03 (d, J = 14.8 Hz, 1H), 2.97 (dd, J = 16.8, 4.4 Hz, 1H), 2.85 – 2.76 (m, 1H), 2.53 (d, J = 15.2 Hz, 1H), 1.96 (dd, J = 12.0, 5.6 Hz, 1H), 1.85 (d, J = 12.8 Hz, 1H), 1.79 – 1.69 (m, 1H), 1.40 – 1.36 (m, 6H), 1.31 (s, 3H), 1.22 (s, 3H).

To a solution of compound 8 (6.7 g，18.0 mmol) in diethyl ether 40 mL and MeOH

40 mL was added with MeONa (4.87 g, 90 mmol) in 0 °C. The reaction mixture was stirred under $N_2$ at room temperature for 2 h and then concentrated. Water 40 mL was added to the residue and extracted with EA (40 mL×3). The combined organic extract was washed with brine, dried over $Na_2SO_4$ and concentrated. Purification by silica gel chromatography (petroleum ether/EA, 3:1 v/v) to give compound 9 (5.1 g, 80%) as a white solid.

To a solution of compound 9 (5.1 g, 14.3 mmol) in toluene 40 mL was added DDQ (4.9 g, 21.5 mmol). The reaction mixture was heated to 100 °C for 3 h and then was quenched with saturated aq. $NaHCO_3$ 50 mL. The two layers were separated and the aqueous was extracted twice with EA. The combined organic extract was washed with brine, dried over $Na_2SO_4$ and concentrated. Purification by silica gel chromatography (petroleum ether/EA, 3:1 v/v) to give compound N1 (3 g, 60%) as a yellow solid. mp: 152-153 °C. 1H NMR (400 MHz, $CDCl_3$) δ 8.35 (s, 1H), 7.55 (s, 1H), 6.93 (s, 1H), 3.93 (s, 3H), 3.85 (s, 3H), 2.96 (dd, J = 16.0, 8.0 Hz, 1H), 2.89 – 2.81 (m, 1H), 2.17 (dd, J = 12.0, 2.0 Hz, 1H), 1.97– 1.85 (m, 2H),,), 1.47 (s, 3H), 1.26 (s, 3H), 1.21 (s, 3H). 13C NMR (100 MHz, $CDCl_3$) δ 197.5, 168.3, 166.2, 157.6, 146.6, 133.4, 127.2, 119.3, 115.3, 114.8, 108.1, 56.5, 52.1, 47.0, 45.1, 41.9, 28.8, 27.9, 27.3, 21.3, 19.0. ESI-HRMS(m/z)[M+Na]+ calcd for $C_{21}H_{23}NNaO_4$，376.1519，found 376.1517.

To a solution of N0 (100 mg, 0.29 mmol) and amines (0.59 mmol) in DCM 10 mL was added EDCI (113.1 mg, 0.59 mmol)，HOBt (79.7 mg, 0.59 mmol)，DMAP (144.2 mg, 1.18 mmol). The reaction mixture was stirred at room temperature for 12 h and quenched with $H_2O$, and extracted with DCM (30 mL×3). The combined organic

extract was washed with brine, dried over $Na_2SO_4$ and concentrated. Purification by silica gel chromatography to afford desired compound. N4, yellow solid (61%)； mp: 300-301 °C. 1H NMR (400 MHz, $CDCl_3$) δ 12.31 (s, 1H), 9.00 (dd, J = 8.0, 1.6 Hz, 1H), 8.89 (dd, J = 4.0, 1.6 Hz, 1H), 8.36 (s, 1H), 8.18 (dd, J = 8.0, 1.2 Hz, 1H), 8.11 (s, 1H), 7.58 (t, J = 8.0 Hz, 1H), 7.53 (dd, J = 8.0, 1.2 Hz, 1H), 7.47 (dd, J = 8.,0 4.0 Hz, 1H), 6.99 (s, 1H), 4.26 (s, 3H), 3.07 (dd, J = 16.0, 4.0 Hz, 1H), 2.99 – 2.94 (m, 1H), 2.23 (dd, J = 12.0, 2.4 Hz, 1H), 2.01 – 1.86 (m, 2H), 1.52 (s, 3H), 1.29 (s, 3H), 1.25 (s, 3H). 13C NMR (100 MHz, CDCl3) δ 197.5, 168.1, 163.0, 156.2, 148.4 146.1, 139.3, 136.3, 135.6, 134.2, 128.3, 128.1, 127.6, 121.7, 121.5, 121.5, 117.4, 115.4, 114.8, 107.4, 56.6, 47.1, 45.2, 41.8, 28.9, 28.0, 27.3, 21.3, 19.1. ESI-HRMS(m/z)[M+Na]+ calcd for $C_{29}H_{27}N_3NaO_3$， 488.1945， found 488.1933.

**Trans-well migration assay.** PANC-1, CAPAN-2 and BXPC-3 cells were seeded in trans-well inserts (8 μm pore size, Millipore), and then mixed with N4 with indicated concentration. After treatment, the top side was wiped and the lower side was fixed and stained by 0.2% crystal violet. The migrated cells were counted and the photos were taken by microscope (Olympus).

**Hematoxylin-eosin (HE).** For HE assay, Samples were immediately fixed in 10% neutral buffered formaldehyde for 24 hours, progressively dehydrated in solutions containing an increasing percentage of ethanol (75, 85, 95 and 100%, v/v) and then embedded into paraffin. 4 μm sections were cut from the paraffin blocks. HE samples

were stained with hematoxylin and eosin to indicate the nucleus and cytoplasm, respectively. Histopathological changes were observed under a light microscope. Photos were taken by Leica photomicroscope.

**Knockdown of STAT3 by shRNA.** The sequence of shRNA were list in Supplementary Table 3. In brief, the shRNAs were inserted into pLKO.1 vector. And they, with the packing plasmids pMD2G and pXPAX2, were co-transfected into 293T cells by Lipofectamine 2000 (Thermo Fisher Scientifific, USA) for 72h. And the harvested lentiviruses were infected into PANC-1 and puromycin with a working concentration of 2 μg/mL was used to screen to obtain stable STAT3 knockdown cells.

## Supplementary Figures

**Supplementary Figure 1**

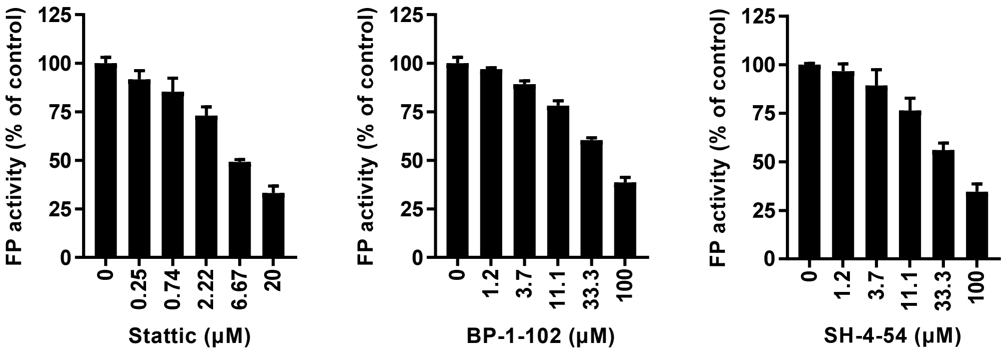


**Supplementary Figure 1. FP assay for Stattic, BP-1-102, SH-4-54.** Purified STAT3 protein was mixed with indicated compounds with increasing concentrations for 1 hour, and then the labeled peptide was added. After 1 hour incubation, the FP signal was measured and $IC_{50}$ was calculated by Graphpad 7.0. Data are representative of three independent determinations.

## Supplementary Figure 2

**A**

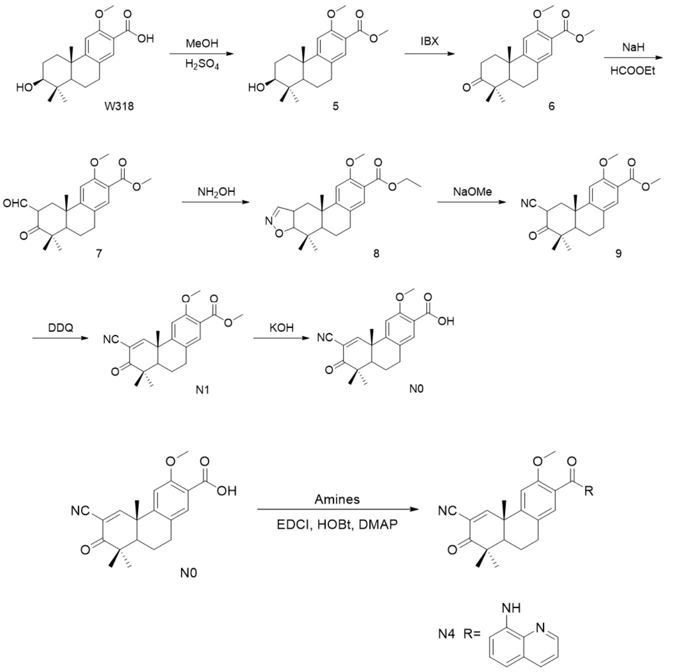


**B**

### Mass Spectrum SmartFormula Report

**Analysis Info**

Analysis Name D:\Data\QWW-20170220\N4_64_01_1267.d
Method tune_wide_for_lc-3min-seg.m
Sample Name N4
Comment

Acquisition Date 2/20/2017 12:19:31 PM
Operator and analy ECNU.CHEM. G.D.Yang
Instrument / Ser# micrOTOF 10293

**Acquisition Parameter**

| | | | | | |
|---|---|---|---|---|---|
| Source Type | ESI | Ion Polarity | Positive | Set Nebulizer | 1.5 Bar |
| Focus | Active | | | Set Dry Heater | 180 °C |
| Scan Begin | 50 m/z | Set Capillary | 4500 V | Set Dry Gas | 6.0 l/min |
| Scan End | 3000 m/z | Set End Plate Offset | -500 V | Set Divert Valve | Waste |

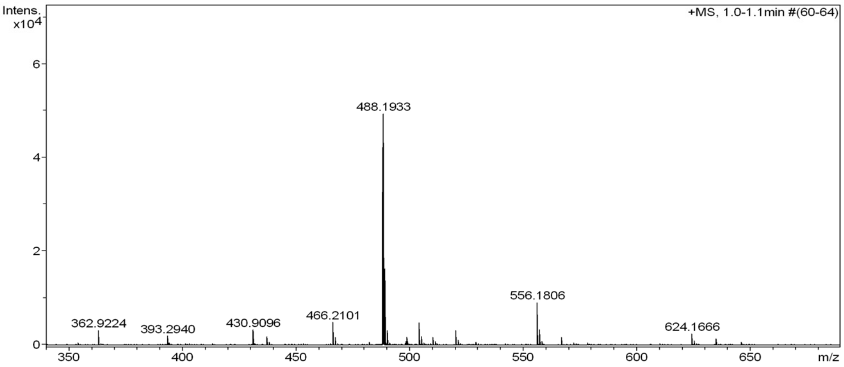


| Meas. m/z | # | Formula | m/z | err [ppm] | Mean err [ppm] | rdb | N-Rule | e⁻ Conf | mSigma | Std I | Std Mean m/z | Std I VarNorm | Std m/z Diff | Std Comb Dev |
|---|---|---|---|---|---|---|---|---|---|---|---|---|---|---|
| 488.1933 | 1 | C 29 H 27 N 3 Na O 3 | 488.1945 | 2.3 | 3.2 | 17.5 | ok | even | 3.72 | 0.0071 | 0.0017 | 0.0028 | 0.0013 | 0.4580 |

**Supplementary Figure 2. (A)** The synthesis scheme of N4. **(B)** The high-resolution mass spectrum (HR MS) of N4.

**Supplementary Figure 3**

**A**

$^{1}H$ NMR

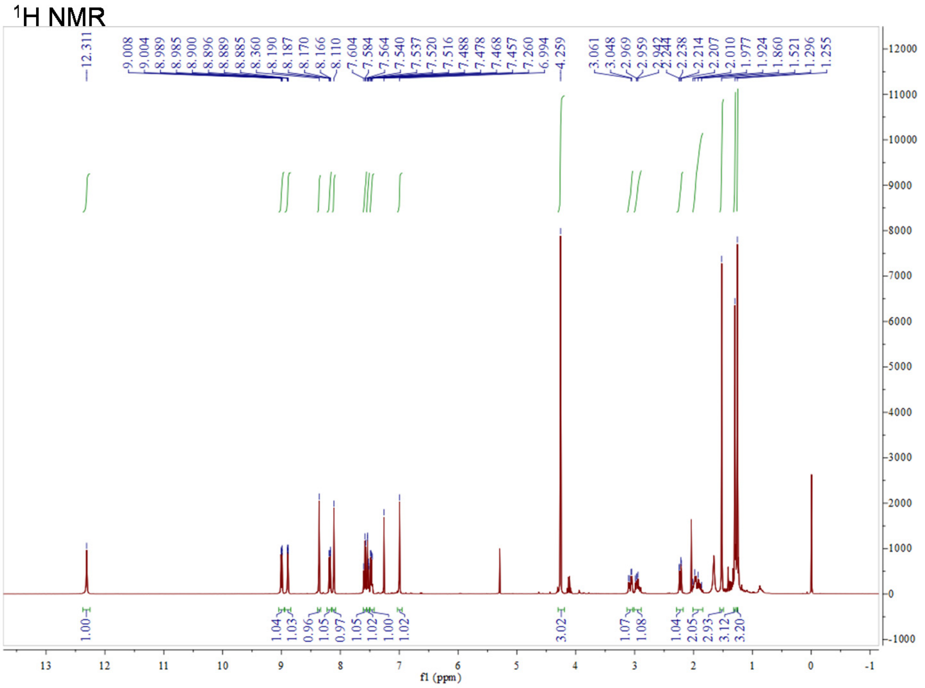


**B**

$^{13}C$ NMR

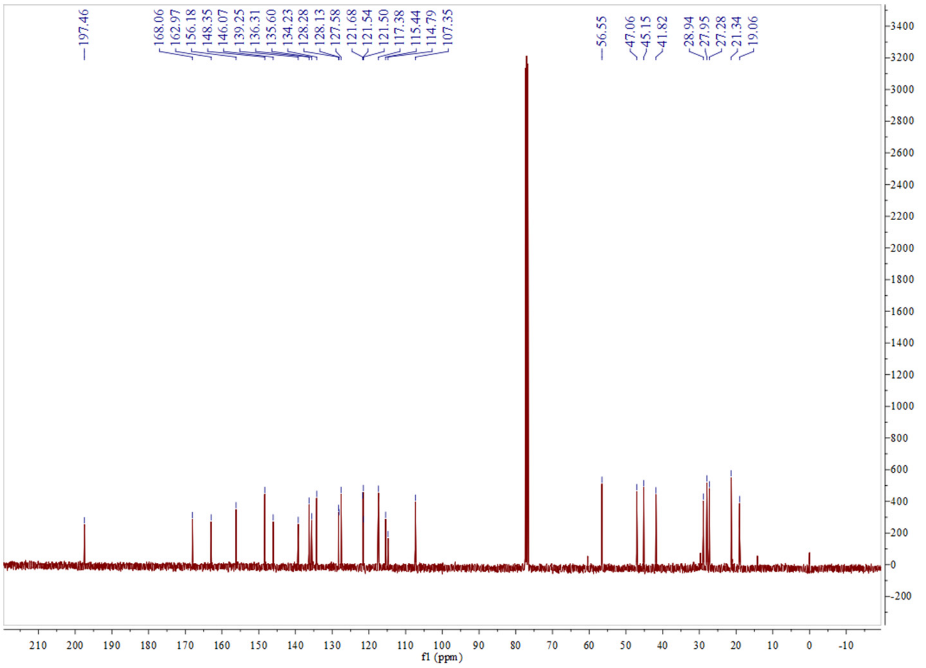


**Supplementary Figure 3. (A)** $^{1}H$-NMR of N4. **(B)** $^{13}C$-NMR of N4.

## Supplementary Figure 4

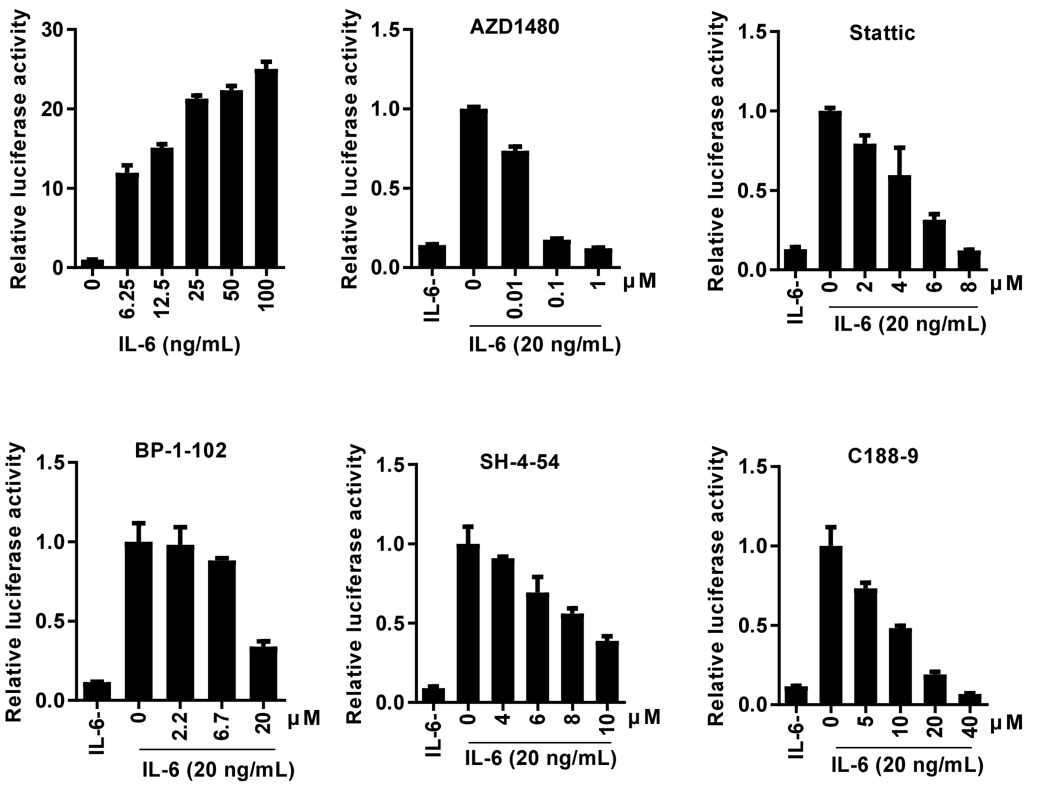


**Supplementary Figure 4. STAT3-response luciferase assay validations.** For luciferase assay, STAT3 response luciferase plasmid was transfected into 293T, IL-6 was an activator of the assay system. Different compounds were added for indicated concentration, luciferase was measured as materials and methods described.

## Supplementary Figure 5

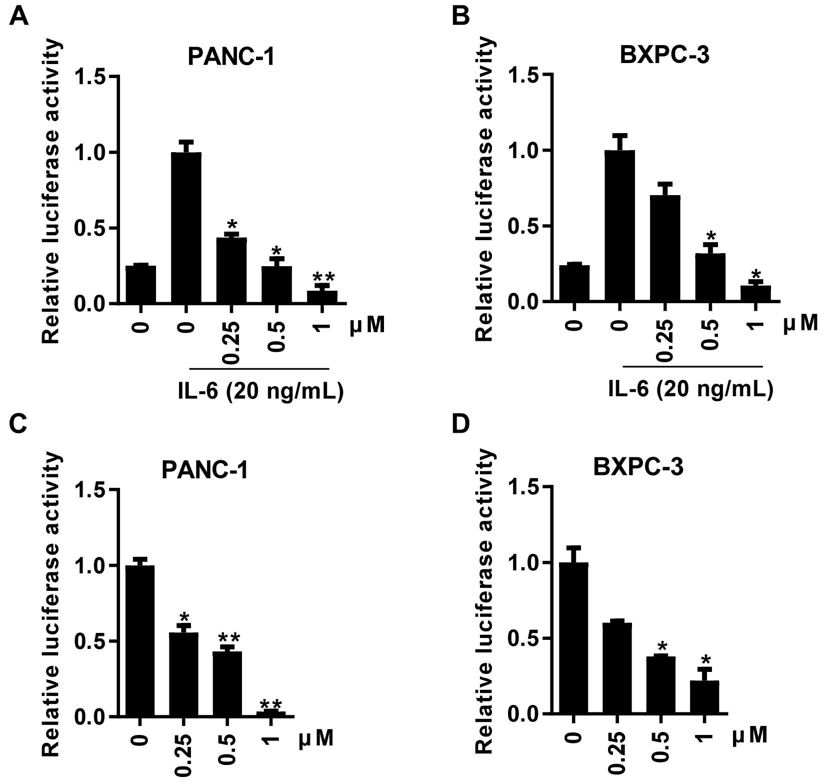


**Supplementary Figure 5. N4 suppresses STAT3-response luciferase activities in PDAC cells. (A-D)** STAT3 specific luciferase reporter plasmid was transiently transfected into PANC-1 (A, C) or BXPC-3 (B, D) cells. After 24 h transfection, IL-6 (20 ng/mL) was added (A, B) or not (C, D). N4 was added at the indicated concentrations. Renilla luciferase activities were used as the internal reference. The values are expressed as mean ± SD and the bars indicate statistically significant differences (t-test), $*P < 0.05$; $**P < 0.01$; $***P < 0.001$.

## Supplementary Figure 6

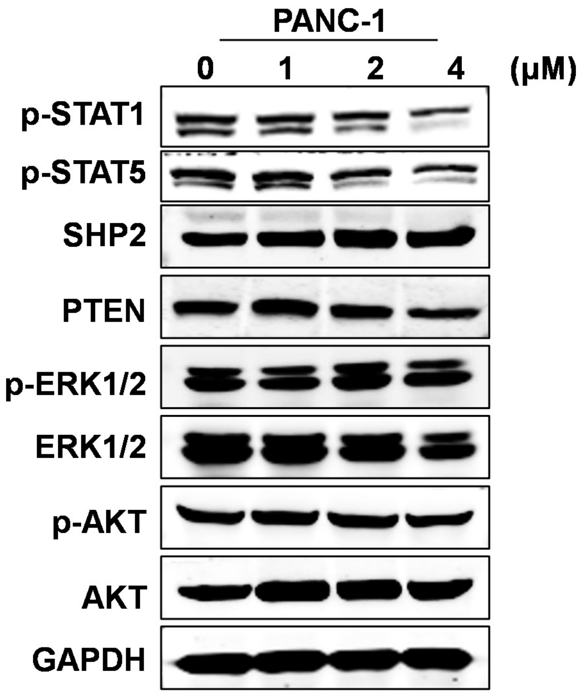


**Supplementary Figure 6. N4 has no obvious influence on the expression of p-STAT1, p-STAT5, SHP2, PTEN, p-AKT, p-ERK1/2 expression.** PANC-1 cells were treated with N4 for 24 hours, then cells were lysed for WB analysis. GAPDH was used as the loading control.

## Supplementary Figure 7

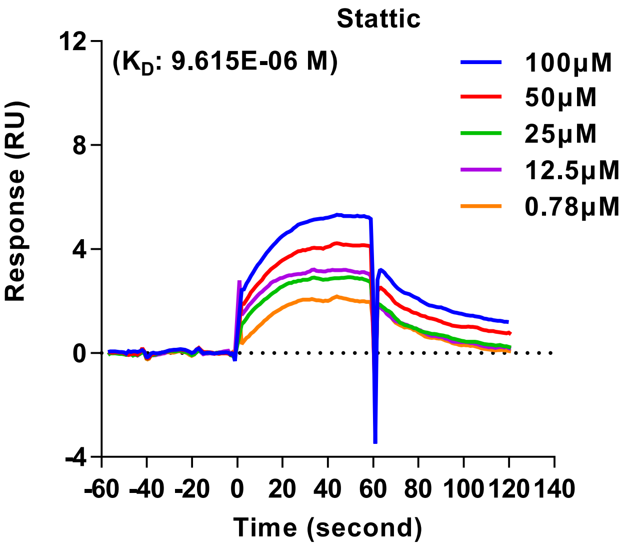


**Supplementary Figure 7.** SPR assay result of Stattic binds to STAT3.

**Supplementary Figure 8**

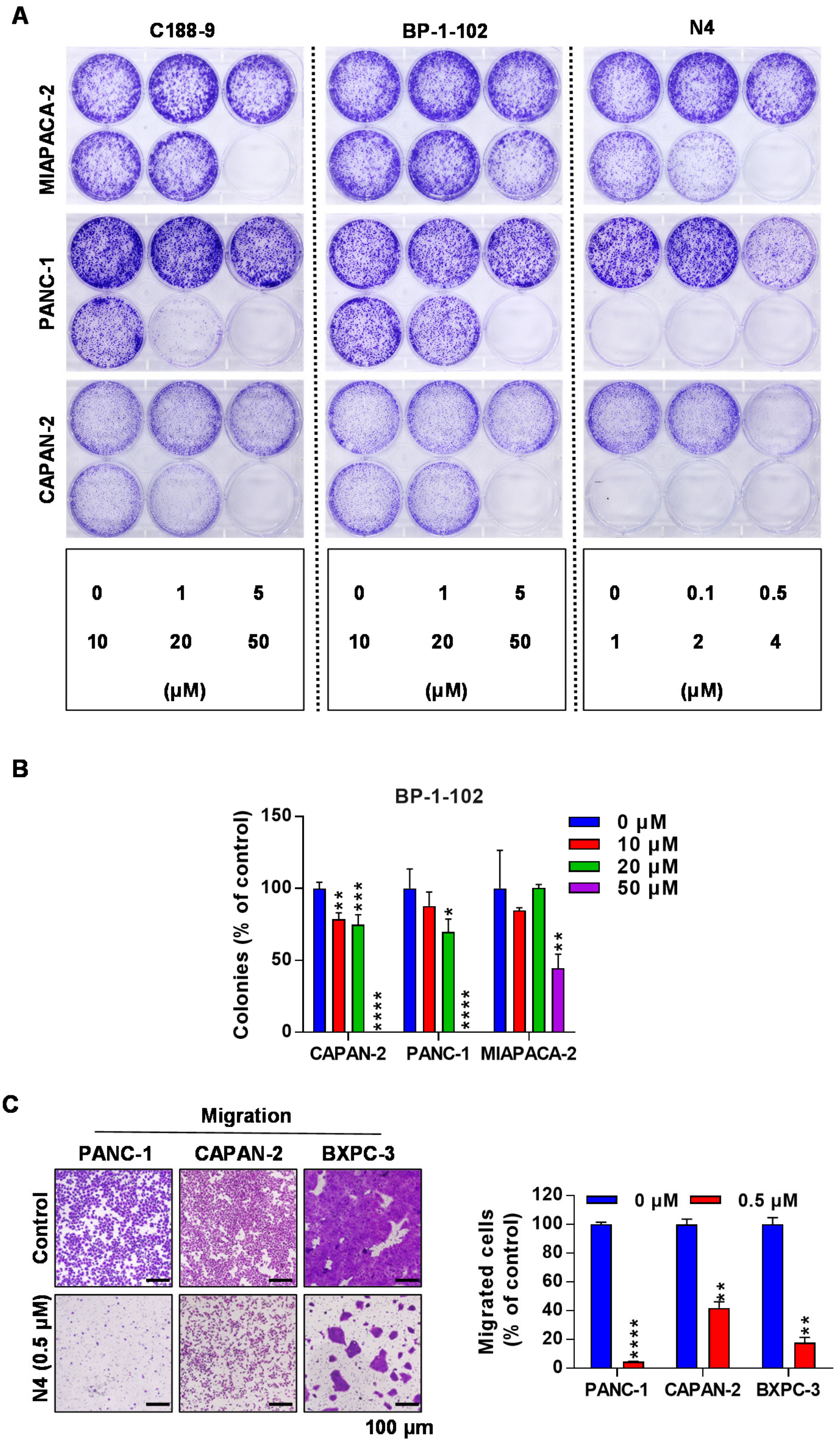


**Supplementary Figure 8. (A)** C188-9, BP-1-102 and N4 suppresses pancreatic cancer cells colony formations. **(B)** Statistic analysis of BP-1-102 on inhibition of CAPAN-2, PANC-1 and MIAPACA-2 cells colony formations. Data shown as mean ± SD of three

independent assays; *P < 0.05, **P < 0.01, ***P < 0.001, ****p < 0.0001 by one-way ANOVA followed by multiple comparisons. **(C)** Pancreatic cancer cells were seeded in the top chamber of Trans-well inserts and treated with N4 the following day. The lower chamber was fixed, stained, and photographed. Experiments were carried out in triplicate with identical results obtained. Data shown as mean ± SD; **P < 0.01, ****p < 0.0001 by Student's t-tests. Scale bar, 100 μm.

Supplementary Figure 9

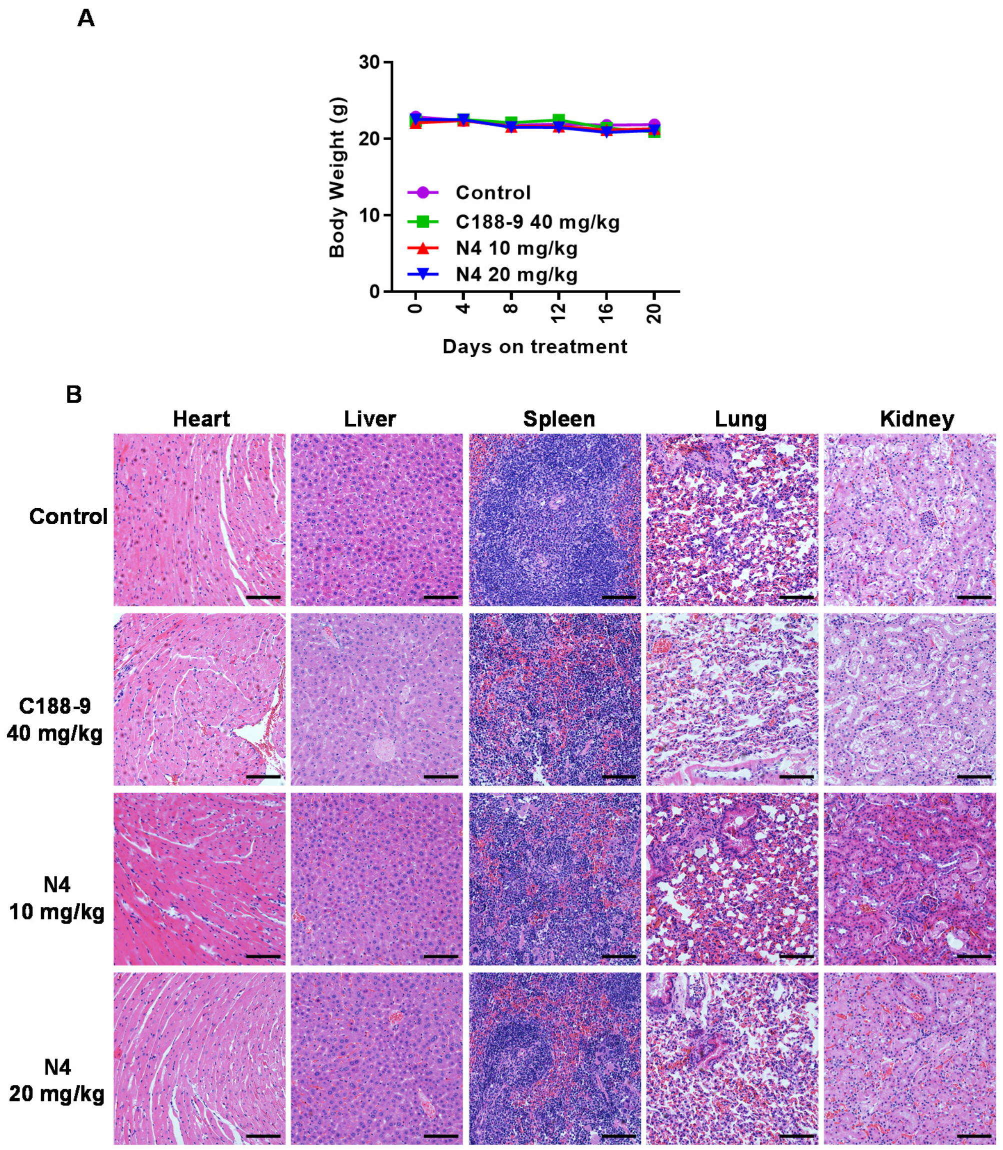


**Supplementary Figure 9. (A)** PANC-1 tumor-bearing mice body weight were measured every 4 days. **(B)** H&E staining of heart, liver, spleen, lung, kidney tissues of vehicle control, C188-9 (40 mg/kg) treatment group and N4 treatment groups. Scale bar, 100μm.

## Supplementary Figure 10

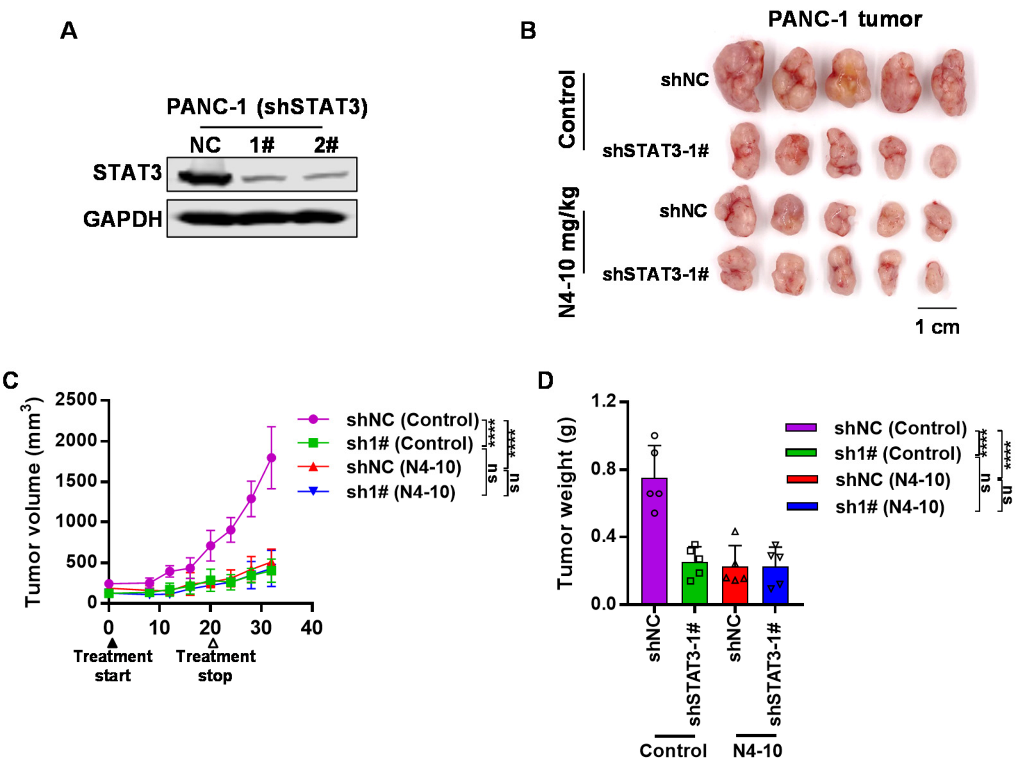


**Supplementary Figure 10. STAT3 knockdown cells are resistant to N4 in vivo. (A)** PANC-1 cells were transfected with shRNA that targeted STAT3 or negative control, and the knockdown efficiency was examined by western blot. **(B)** Mice bearing the PANC-1 cells within STAT3 stable knockdown (shSTAT3-1#) or negative control (shNC), and administration N4 or vehicle (control) every day by *i.p.* After 20 days treatment, the N4 and vehicle were removed for another 12 days. Then the mice were sacrificed and the tumor was removed and photographed (n=5 per group). **(C)** The tumor volume was measured every 3-4 days and the volume was calculated by the formula: length × width$^2$ ×0.52, data are shown as mean ± SD, ****P < 0.0001 and n.s., not significant by one-way ANOVA followed by multiple-comparison tests. **(D)** After the experiments, all the tumors were weighed, and these data are expressed as mean ± SD; ****P < 0.0001 and n.s., not significant by one-way ANOVA.

## Supplementary Tables

**Supplementary Table 1.** Chemical structure of the N-series family, Structure-activity relationship for the derivative analogs and $IC_{50}$ value for compounds inhibited the STAT3 dimerization in FP assay.

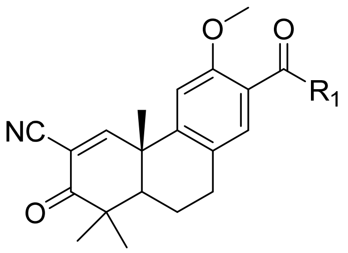


| Code | R1 | FP assay ($IC_{50}$ μM) | Code | R1 | FP assay ($IC_{50}$ μM) |
|---|---|---|---|---|---|
| **N0** | **OH** | **>20** | **N25** | 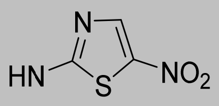 | **1.01** |
| **N1** | O— | **15.6** | **N26** | 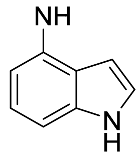 | **1.88** |
| **N2** | 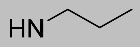 | **7.71** | **N29** | 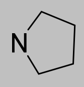 | **8.96** |
| **N3** | 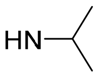 | **8.23** | **N30** | 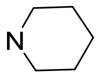 | **6.14** |
| **N4** | 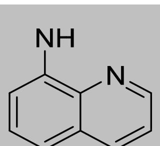 | **0.56** | **N31** | 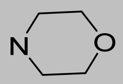 | **20** |
| **N5** | 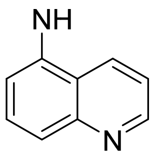 | **8.41** | **N32** | 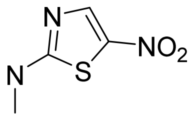 | **5.37** |

| | | | | | |
|---|---|---|---|---|---|
| **N17** | 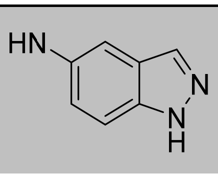 | **>20** | **N33** | 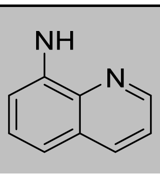 | **>20** |
| **N18** | 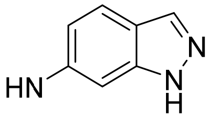 | **6.79** | **N34** | 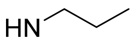 | **>20** |
| **N19** | 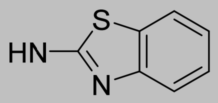 | **1.45** | **N35** | 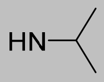 | **>20** |
| **N20** | 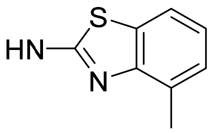 | **8.33** | **N37** | 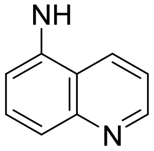 | **>20** |
| **N21** | 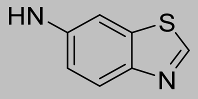 | **4.58** | **STATTIC** | **-** | **4.99** |
| **N22** | 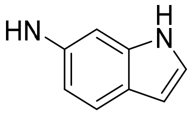 | **6.38** | **BP-1-102** | **-** | **34.85** |
| **N24** | 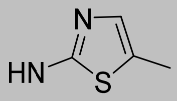 | **8.73** | **SH-4-54** | **-** | **30.43** |

**Supplementary Table 2. Sequence of siRNA**

| Name of Gene | si-RNA (5' to 3') |
|---|---|
| STAT3 siRNA #1 | GCGUCCAGUUCACUACUAATT |
| STAT3 siRNA#2 | GCAACAGAUUGCCUGCAUUTT |
| Control siRNA | AATTCTCCGAACGTGTCACGT(dTdT) |

**Supplementary Table 3. Sequence of shRNA**

| Name of Gene | shRNA (5' to 3') |
|---|---|
| STAT3 shRNA #1 | GCACAATCTACGAAGAATC |
| STAT3 shRNA#2 | GGCGTCCAGTTCACTACTA |
| Control shRNA | CAACAAGATGAAGAGCACC |
