# Supplementary material for: Targeting STAT3 by a small molecule suppresses pancreatic cancer progression
Source: Oncogene. 2021 Jan 8;40(8):1440–57. doi: 10.1038/s41388-020-01626-z (PMC7906907; doi:10.1038/s41388-020-01626-z)
Supplement: Supplementary file 2 — All of the co-authors email responses for the manuscript authorship [file 41388_2020_1626_MOESM2_ESM.pdf]

Mail：

Dear all authors:

Please note that you are listed as a co-author on the manuscript "Targeting STAT3 by a small molecule suppresses pancreatic cancer progression" (reference number: ONC-2020-00196RRR), which has been provisionally accepted for publication in Oncogene. For the final acceptance for this paper, please ensure the authorship, and reply to this email confirming that you agree to the author list. The detailed author list are: Huang Chen[1#], Aiwu Bian[1#], Lian-fang Yang[2], Xuan Yin[3], Jie Wang[2], Chaowen Ti[1], Ying Miao[1], Shihong Peng[1], Shifen Xu[3], Mingyao Liu[1*], Wen-Wei Qiu[2*] and Zhengfang Yi[1, 2*].

Thank you very much.

Sincerely,

Zhengfang Yi

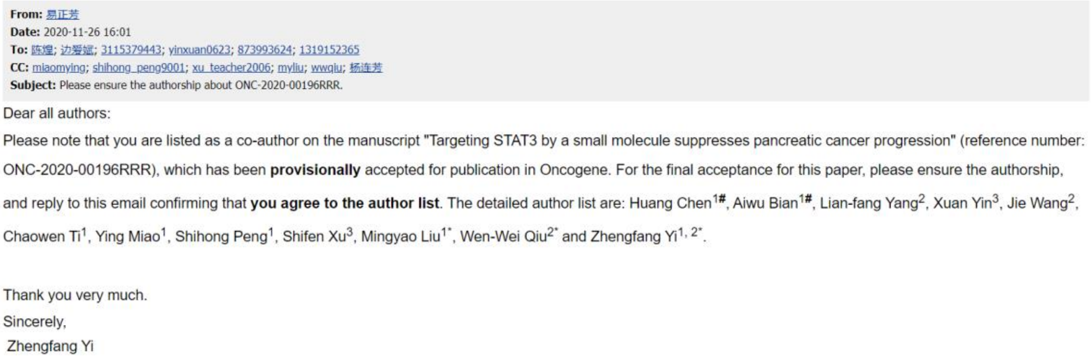

From: 易正芳

Date: 2020-11-26 16:01

To: 陈煌; 边爱娬; 3115379443; yinxuan0623; 873993624; 1319152365

CC: miaomying; shihong_peng9001; xu_teacher2006; myliu; wwqiu; 杨连芳

Subject: Please ensure the authorship about ONC-2020-00196RRR.

Dear all authors:

Please note that you are listed as a co-author on the manuscript "Targeting STAT3 by a small molecule suppresses pancreatic cancer progression" (reference number: ONC-2020-00196RRR), which has been **provisionally** accepted for publication in Oncogene. For the final acceptance for this paper, please ensure the authorship, and reply to this email confirming that **you agree to the author list**. The detailed author list are: Huang Chen[1#], Aiwu Bian[1#], Lian-fang Yang[2], Xuan Yin[3], Jie Wang[2], Chaowen Ti[1], Ying Miao[1], Shihong Peng[1], Shifen Xu[3], Mingyao Liu[1*], Wen-Wei Qiu[2*] and Zhengfang Yi[1, 2*].

Thank you very much.

Sincerely,

Zhengfang Yi

Response of all author:

Huang Chen:

Re:Please ensure the authorship about ONC-2020-00196RRR.

发件人：陈煌<huangchen9104@163.com>

收件人：易正芳<zfyi@bio.ecnu.edu.cn>

抄送人：边爱斌<bian_aiwu@163.com> 3115379443<3115379443@qq.com> yinxuan0623<yinxuan0623@hotmail.com>
1319152365<1319152365@qq.com> ↓还有6个联系人 保存所有抄送人

时 间：2020年11月26日 16:08 (星期四)

Dear professor Yi:

I agree with the authorship of this manuscript.

Thank You very much.

Huang Chen

Aiwu Bian:

Re: Please ensure the authorship about ONC-2020-00196RRR.

发件人：边爱斌<bian_aiwu@163.com>

收件人：易正芳<zfyi@bio.ecnu.edu.cn>

抄送人：陈煌<huangchen9104@163.com> 3115379443@qq.com<3115379443@qq.com>
提超文<1319152365@qq.com> ↓还有6个联系人 保存所有抄送人

时 间：2020年11月26日 16:12 (星期四)

Dear professor Yi:

I agree with the authorship of this manuscript.

Thank You very much.

Aiwu Bian

Lian-fang Yang:

回复：Please ensure the authorship about ONC-2020-00196RRR.

发件人：杨<3115379443@qq.com>

收件人：易正芳<zfyi@bio.ecnu.edu.cn> 陈煌<huangchen9104@163.com> 边爱斌<bian_aiwu@163.com>

个联系人 保存所有收件人

时 间：2020年11月26日 17:56 (星期四)

Dear professor Yi: I agree with the authorship of this manuscript. Thank You very much. Lianfang Yang

Xuan Yin:

Re: Please ensure the authorship about ONC-2020-00196RRR.

发件人：殷 萱<yinxuan0623@hotmail.com>

收件人：易正芳<zfyi@bio.ecnu.edu.cn>

时 间：2020年11月26日 16:26 (星期四)

Dear Professor Yi:

I agree with the authorship of this manuscript.

Thank You very much.

Xuan Yin

Jie Wang:

回复：Please ensure the authorship about ONC-2020-00196RRR.

发件人：王杰<873993624@qq.com>

收件人：易正芳<zfyi@bio.ecnu.edu.cn>

时 间：2020年11月26日 17:13 (星期四)

Dear professor Yi:

I agree with the authorship of this manuscript.

Thank You very much.

Jie Wang

Ying Miao:

Re:Please ensure the authorship about ONC-2020-00196RRR.

发件人：miaomying<miaomying@163.com>

收件人：易正芳<zfyi@bio.ecnu.edu.cn>

时　间：2020年11月27日 13:19 (星期五)

Dear professor Yi:

I agree with the authorship of this manuscript.

Thank You very much.

Ying Miao

Chaowen Ti:

回复：Please ensure the authorship about ONC-2020-00196RRR.

发件人：提超文<1319152365@qq.com>

收件人：易正芳<zfyi@bio.ecnu.edu.cn> 陈煌<huangchen9104@163.com> 边爱斌<bian_aiwu@1

1个联系人　保存所有收件人

抄送人：miaomying<miaomying@163.com> shihong_peng9001<shihong_peng9001@yeah.net> x

wwqiu<wwqiu@chem.ecnu.edu.cn> ↓还有1个联系人　保存所有抄送人

时　间：2020年11月26日 16:24 (星期四)

Dear professor Yi:

I agree with the authorship of this manuscript.

Thank You very much.

Chaowen Ti

Shihong Peng:

Re: Please ensure the authorship about ONC-2020-00196RRR.

发件人：彭世鸿<shihong_peng9001@yeah.net>

收件人：易正芳 老师<zfyi@bio.ecnu.edu.cn>

抄送人：陈煌<huangchen9104@163.com> 边爱斌<bian_aiwu@163.com> 3115379443@qc

873993624@qq.com<873993624@qq.com> ↓还有6个联系人 保存所有抄送人

时 间：2020年11月26日 16:10 (星期四)

Dear professor Yi:

I agree with the authorship of this manuscript.

Thank you very much.

Shihong Peng, Ph.D

Shifen Xu:

Re:Please ensure the authorship about ONC-2020-00196RRR.

发件人：peony<xu_teacher2006@126.com>

收件人：易正芳<zfyi@bio.ecnu.edu.cn>

抄送人：陈煌<huangchen9104@163.com> 边爱斌<bian_aiwu@163.com> 3115379443<3115379443@qq.com> yinxuar

↓还有6个联系人 保存所有抄送人

时 间：2020年11月27日 12:44 (星期五)

Dear Professor Yi:
I agree with the authorship of this manuscript.

Thank you very much

--

Shifen Xu MD & PhD

Mingyao Liu:

Re:Please ensure the authorship about ONC-2020-00196RRR.

发件人: 刘明耀<myliu@bio.ecnu.edu.cn>

收件人: 易正芳<zfyi@bio.ecnu.edu.cn>

时　间: 2020年11月30日 10:33 (星期一)

Dear professor Yi:

I agree with the authorship of this manuscript.

Thank You very much.

Mingyao Liu

Wen-Wei Qiu:

发件人: wwqiu@chem.ecnu.edu.cn<wwqiu@chem.ecnu.edu.cn>

收件人: zfyi<zfyi@bio.ecnu.edu.cn> huangchen9104<huangchen9104@163.com> yinxuan0623<yinxuan0623@hotmail.com> ↓还有2个联系人 保存所有收件人

抄送人: miaomying<miaomying@163.com> 彭世鸿<shihong_peng9001@yeah.net> 1198756058<1198756058@qq.com>

时　间: 2020年11月27日 14:27 (星期五)

Dear professor Yi:

I agree with the authorship of this manuscript.

Thank You very much.

Wen-Wei Qiu
